# DNA hypermethylation appears early and shows increased frequency with dysplasia in Lynch syndrome-associated colorectal adenomas and carcinomas

**DOI:** 10.1186/s13148-015-0102-4

**Published:** 2015-07-22

**Authors:** Satu Valo, Sippy Kaur, Ari Ristimäki, Laura Renkonen-Sinisalo, Heikki Järvinen, Jukka-Pekka Mecklin, Minna Nyström, Päivi Peltomäki

**Affiliations:** Division of Genetics, Department of Biosciences, University of Helsinki, Helsinki, Finland; Department of Medical and Clinical Genetics, University of Helsinki, Helsinki, Finland; Genome-Scale Biology, Research Programs Unit, University of Helsinki, Helsinki, Finland; Department of Pathology, HUSLAB, Helsinki University Central Hospital and University of Helsinki, Helsinki, Finland; Department of Gastrointestinal and General Surgery, Helsinki University Central Hospital, Helsinki, Finland; Department of Surgery, Jyväskylä Central Hospital, University of Eastern Finland, Jyväskylä, Finland

**Keywords:** Lynch syndrome, Colorectal cancer, Adenoma-carcinoma sequence, DNA methylation, CpG island methylator phenotype, MS-MLPA

## Abstract

**Background:**

Lynch syndrome (LS) is associated with germline mutations in DNA mismatch repair (MMR) genes. The first “hit” to inactivate one allele of the predisposing MMR gene is present in every cell, contributing to accelerated tumorigenesis. Less information is available of the nature, timing, and order of other molecular “hits” required for tumor development. To this end, MMR protein expression and coordinated promoter methylation were examined in colorectal specimens prospectively collected from LS mutation carriers (*n* = 55) during colonoscopy surveillance (10/2011–5/2013), supplemented with retrospective specimens.

**Results:**

Loss of MMR protein corresponding to the gene mutated in the germline increased with dysplasia, with frequency of 0 % in normal mucosa, 50–68 % in low-grade dysplasia adenomas, and 100 % in high-grade dysplasia adenomas and carcinomas. Promoter methylation as a putative “second hit” occurred in 1/56 (2 %) of tumors with silenced MMR protein. A general hypermethylation tendency was evaluated by two gene sets, eight CpG island methylator phenotype (CIMP) genes, and seven candidate tumor suppressor genes linked to colorectal carcinoma (CRC). Hypermethylation followed the same trend as MMR protein loss and was present in some low-grade dysplasia adenomas that still expressed MMR protein suggesting the absence of a “second hit.” To assess prospectively collected normal mucosa for carcinogenic “fields,” the specimen donors were stratified according to age at biopsy (50 years or below vs. above 50 years) and further according to the absence vs. presence of a (previous or concurrent) diagnosis of CRC. In mutation carriers over 50 years old, two markers from the candidate gene panel (*SFRP1* and *SLC5A8*) revealed a significantly elevated average degree of methylation in individuals with CRC diagnosis vs. those without.

**Conclusions:**

Our findings emphasize the importance and early appearance of epigenetic alterations in LS-associated tumorigenesis. The results serve early detection and assessment of progression of CRC.

**Electronic supplementary material:**

The online version of this article (doi:10.1186/s13148-015-0102-4) contains supplementary material, which is available to authorized users.

## Background

Colorectal cancer (CRC) develops via multiple steps involving genetic and epigenetic changes. The majority of CRCs are sporadic. In Lynch syndrome (LS), inherited defects of the DNA mismatch repair (MMR) genes *MLH1*, *MSH2*, *MSH6*, and *PMS2* confer high lifetime risks of CRC and extracolonic cancers [1]. Cancers arising in LS mutation carriers as well as some 12 % of sporadic CRCs exhibit microsatellite instability (MSI) [2]. Promoter methylation of *MLH1* was recognized as a primary cause for sporadic MSI CRC [3, 4]. In LS, germline mutation combined with somatic mutations or loss of heterozygosity underlies biallelic inactivation of MMR genes. MMR defects together with other genetic and epigenetic changes accelerate neoplastic transformation of the normal colonic epithelium [5].

Aberrant CpG island methylation affecting multiple tumor suppressor genes is frequent in sporadic CRC and colonic adenomas [6, 7], giving rise to a CpG island methylator phenotype (CIMP). CIMP tumors form a subtype with distinct histology compared to tumors derived from the traditional adenoma carcinoma sequence [8]. The molecular basis of CIMP and its clinical implications are only beginning to be explored [5, 9]. In hereditary CRC, the importance of CIMP is largely unknown.

This investigation was undertaken to clarify the role of CIMP and its time of appearance in colorectal adenoma-carcinoma progression sequence in LS. LS mutation carriers are enrolled in lifelong colonoscopy surveillance with 2–3-year intervals [10], and we took advantage of the regular surveillance to obtain consecutive specimens. Furthermore, a previous mouse study implicated a number of candidate genes in association with *MLH1* mutation and diet [11], prompting us to evaluate the respective genes as methylation targets in human LS.

## Results

### Study design

Biopsy specimens fresh frozen from different parts of the colon and rectum, together with blood samples, were obtained from 55 consecutive LS mutation carriers who underwent regular colonoscopy screening or colectomy at two Finnish hospitals during 10/2011–5/2013 (Table 1). This prospective series was used to study DNA methylation changes in normal colonic mucosa with respect to aging and previously diagnosed cancer. As only a few individuals developed colorectal lesions (mostly hyperplastic polyps) during the 1.5-year interval, all archival tubular and villous adenomas and carcinomas previously diagnosed in the same individuals were gathered (retrospective series in Table 1) and used to investigate DNA methylation changes occurring in the adenoma carcinoma progression sequence. Colonic and rectal biopsies from 22 familial adenomatous polyposis (FAP) mutation carriers who participated in colonoscopy screening (Table 1) were studied for comparison.

**Table 1:** Characteristics of sample series

	Prospective series	Retrospective series
	No. of individuals or specimens	No. of individuals or specimens
Lynch syndrome		
• Individuals with mutation in		
*MLH1*	39 (71 %)	33 (77 %)
*MSH2*	13 (24 %)	7 (16 %)
*MSH6*	3 (5 %)	3 (7 %)
Total	55	43
• Colorectal specimens		
Normal colonic mucosa	55	24
Hyperplastic polyp	10	–
Low dysplasia adenoma	5	27
High dysplasia adenoma	3	13
Carcinoma	3	20
Total	76	84
Familial adenomatous polyposis		
• Individuals with mutation in		
*APC*	22 (100 %)	–
• Colorectal specimens		
Normal colonic mucosa	22	–
Adenoma (all dysplasia grades)	23	–
Carcinoma	–	–
Total	45	–

Note: three carcinomas, three low-grade dysplasia adenomas, and two high-grade dysplasia adenomas were available as fresh frozen and FFPE samples and were simultaneously included in both prospective and retrospective series

### MMR status and analysis of MMR gene promoter methylation as the “second hit”

To test if the predisposing MMR gene had undergone somatic inactivation of the remaining wild-type allele, colorectal specimens were evaluated for MMR protein expression by immunohistochemical (IHC) analysis. All adenomas with high-grade dysplasia and all carcinomas from LS patients showed loss of MMR protein corresponding to the gene mutated in the germline whereas only 68 %, 67 % and 50 % of adenomas with low-grade dysplasia showed loss of expression in *MLH1*, *MSH2*, and *MSH6* mutation carriers, respectively (Table 2). The difference (adenomas with low-grade dysplasia vs. adenomas with high-grade dysplasia and adenomas with low-grade dysplasia vs. carcinomas) was statistically significant for *MLH1*-associated tumors. Overall, the results suggest that silencing of the relevant MMR protein expression is a relatively late event in LS tumorigenesis. MSI analysis with the mononucleotide repeat markers *BAT25* and *BAT26* showed that all adenomas and carcinomas with absent MMR protein were microsatellite unstable with one exception, an adenoma with low-grade dysplasia from a *MSH2* mutation carrier. The low tumor cell percentage (20 %) in that particular sample was likely to explain stable microsatellites. All eight low-grade dysplasia adenomas retaining MMR protein expression were microsatellite stable.

**Table 2:** Proportion of decreased MMR protein expression in Lynch syndrome adenomas and carcinomas

	Proportion (%) with decreased MMR protein corresponding to the gene mutated in germline											
	MLH1	*p* value vs. normal colon	*p* value vs. low dysplasia	MSH2	*p* value vs. normal colon	*p* value vs. low dysplasia	MSH6	*p* value vs. normal colon	*p* value vs. low dysplasia	Total	*p* value vs. normal colon	*p* value vs. low dysplasia
Normal colon	0/33 (0 %)			0/7 (0 %)			0/3 (0 %)			0/43 (0 %)		
Adenoma low dysplasia	17/25 (68 %)	<0.0001		4/6 (67 %)	0.021		1/2 (50 %)	NS		22/33 (67 %)	<0.0001	
Adenoma high dysplasia	13/13 (100 %)	<0.0001	0.034	1/1 (100 %)	NS	NS	–		NS	14/14 (100 %)	<0.0001	0.020
Carcinoma	12/12 (100 %)	<0.0001	0.036	3/3 (100 %)	0.008	NS	5/5 (100 %)	0.018	NS	20/20 (100 %)	<0.0001	0.003

NS, no statistical significance

**Fig. 1:**
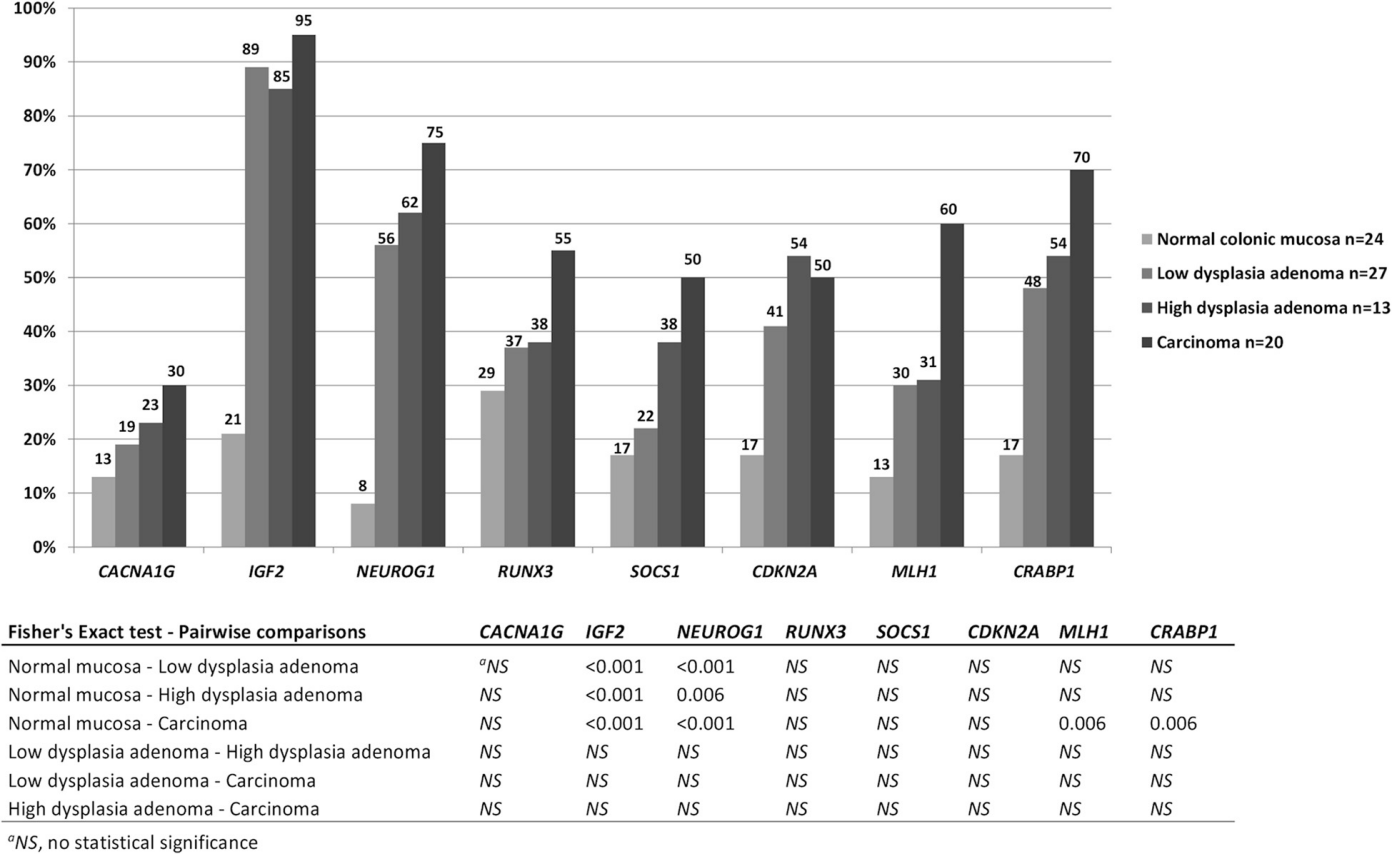
Frequency of hypermethylated CIMP markers in the LS retrospective series. Numerical values of percentages are given above each bar. Hypermethylation thresholds were calculated according to stringency level I. Pairwise comparisons were calculated by Fisher’s exact test (two-sided *p* values), and the *p* values were adjusted for multiple comparisons by Bonferroni correction

Promoter methylation as a possible “second hit” was assessed in tumors lacking MMR protein. Methylation of *MLH1* was mostly detected in the distal promoter (region A [12]). This region was methylated in 30 %, 31 % and 60 % of adenomas with low-grade dysplasia, adenomas with high-grade dysplasia and carcinomas, respectively (Fig. 1). On the contrast, methylation of the proximal promoter (region C), most commonly associated with MLH1 protein loss, was only observed in one adenoma having high-grade dysplasia. No methylation was detected in promoter regions of *MSH2* or *MSH6*. Taken together, promoter methylation constituted a putative “second hit” in 1/56 (2 %) tumors with silenced MMR protein.

### CIMP

To assess whether coordinated methylation of multiple CpG islands that are normally unmethylated plays a role in colorectal tumorigenesis, methylation-specific multiplex ligation-dependent probe amplification (MS-MLPA) was used to study eight genes firmly associated with CIMP (*CACNA1G*, *IGF2*, *NEUROG1*, *RUNX3*, *SOCS1*, *CDKN2A*, *MLH1*, and *CRABP1*). The MS-MLPA CIMP probe mix contains 3–6 probes for each CIMP marker gene, and the average methylation dosage ratios (Dm) obtained for each probe and type of specimen are given in Additional file 1: Table S1. Hypermethylation in tumor tissues was evaluated relative to probe-specific thresholds derived from normal mucosa. The hypermethylation thresholds for the retrospective (FFPE) series are given in Additional file 2: Table S2 and those for the prospective (fresh frozen) series in the legend of Additional file 3: Figure S1. A gene was considered hypermethylated when at least one fourth (25 %) or more of probe target sites were methylated [13].

The prospective LS series revealed increased methylation in adenomas and carcinomas vs. normal colonic mucosa for the CIMP markers *IGF2*, *NEUROG1*, and *CRABP1* (*p* values mostly non-significant due to small numbers of specimens; Additional file 3: Figure S1A). Hyperplastic polyps, too, showed frequent hypermethylation with *IGF2* and *NEUROG1*. LS vs. FAP-associated adenomas and matching normal mucosa showed comparable frequencies of hypermethylation.

The analyses were extended to the retrospective LS series with higher number of tumors available (Fig. 1). Adenomas with high-grade dysplasia and carcinomas showed the highest frequencies of hypermethylation (defined with stringency level I of Additional file 2: Table S2). The frequencies of hypermethylation for *IGF2* and *NEUROG1* were significantly increased in all tumor types when compared to normal colon. Notably, the difference was significant already in adenomas with low-grade dysplasia (89 % vs. 21 %, *p* < 0.001, for *IGF2* and 56 % vs. 8 %, *p* < 0.001, for *NEUROG1*). Only carcinomas showed significantly elevated hypermethylation frequencies for *MLH1* and *CRABP1*.

**Fig. 2:**
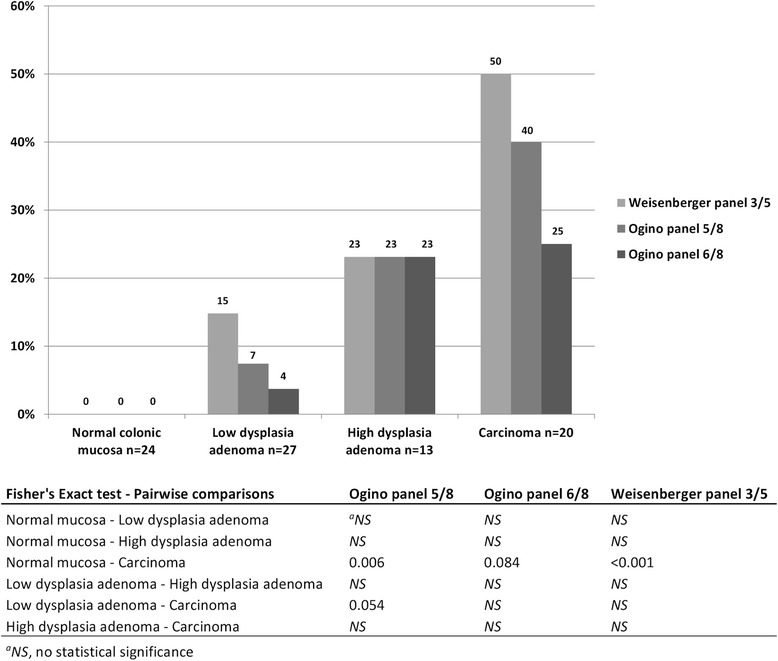
Frequency of CIMP(+) specimen calculated by three different criteria. Numerical values of percentages are given above each bar. Hypermethylation thresholds were calculated according to stringency level II. Pairwise comparisons were calculated by Fisher’s exact test (two-sided *p* values), and the *p* values were adjusted for multiple comparisons by Bonferroni correction

As no consensus exists on how to score CIMP and no single panel is superior to others [13], three different marker panels were considered. Colorectal specimens from the retrospective series were divided into CIMP(+) or CIMP(−) categories by using the Ogino 5/8, Ogino 6/8, and Weisenberger 3/5 panels (“Methods” section). Here, stringency level II (Additional file 2: Table S2) was used to calculate probe-specific thresholds to avoid hypermethylation in normal mucosa. The frequency of CIMP(+) specimens increased from normal mucosa to adenomas with low-grade dysplasia to adenomas with high-grade dysplasia to carcinomas regardless of the marker system used (Fig. 2). The Weisenberger panel yielded somewhat higher CIMP(+) frequencies compared to the Ogino panels. Accordingly, 15 %, 23 % and 50 % of the adenomas with low-grade dysplasia, adenomas with high-grade dysplasia, and carcinomas, respectively, were CIMP(+) when using the Weisenberger 3/5 panel, compared to 7 %, 23 % and 40 % with the Ogino 5/8 criteria, and 4 %, 23 % and 25 % with the Ogino 6/8 criteria (Fig. 2). Formal statistical significance was reached in the normal mucosa vs. carcinomas comparison according to Ogino 5/8 (*p* = 0.006) and Weisenberger 3/5 (*p* < 0.001) criteria and borderline significance in the adenomas with low-grade dysplasia vs. carcinomas comparison according to Ogino 5/8 (*p* = 0.054) criteria. CIMP(+) vs. CIMP(−) tumors were diagnosed at similar average ages (48 vs. 49 years for adenomas and 49 vs. 48 years for carcinomas) excluding age as a possible confounding factor in the analyses (see below).

### Methylation analysis of candidate genes

A custom MS-MLPA kit was designed to study methylation of seven candidate genes previously associated with early colon oncogenesis in an experimental mouse model (*DKK1*, *SFRP1*, *SFRP2*, *SFRP5*, *CDH1*, *HOXD1*, and *SLC5A8* (Additional file 4: Table S3) [11]. The average Dm obtained for each probe and type of specimen are shown in Additional file 1: Table S1. Probe-specific hypermethylation thresholds were determined as described for CIMP markers (stringency level I), separately for the retrospective series (Additional file 2: Table S2) and the prospective series (legend of Additional file 3: Figure S1).

The prospective LS series indicated significantly higher frequencies of hypermethylation for *SFRP1* (95 %, *p* = 0.006) and *SFRP2* (67 %, *p* = 0.012) in carcinomas vs. normal colonic mucosa (Additional file 3: Figure S1B). Additionally, 50 % of hyperplastic polyps revealed hypermethylation for *SFRP1* relative to normal mucosa (*p* = 0.030). Hypermethylation frequencies in colonic tissues were comparable in LS vs. FAP.

**Fig. 3:**
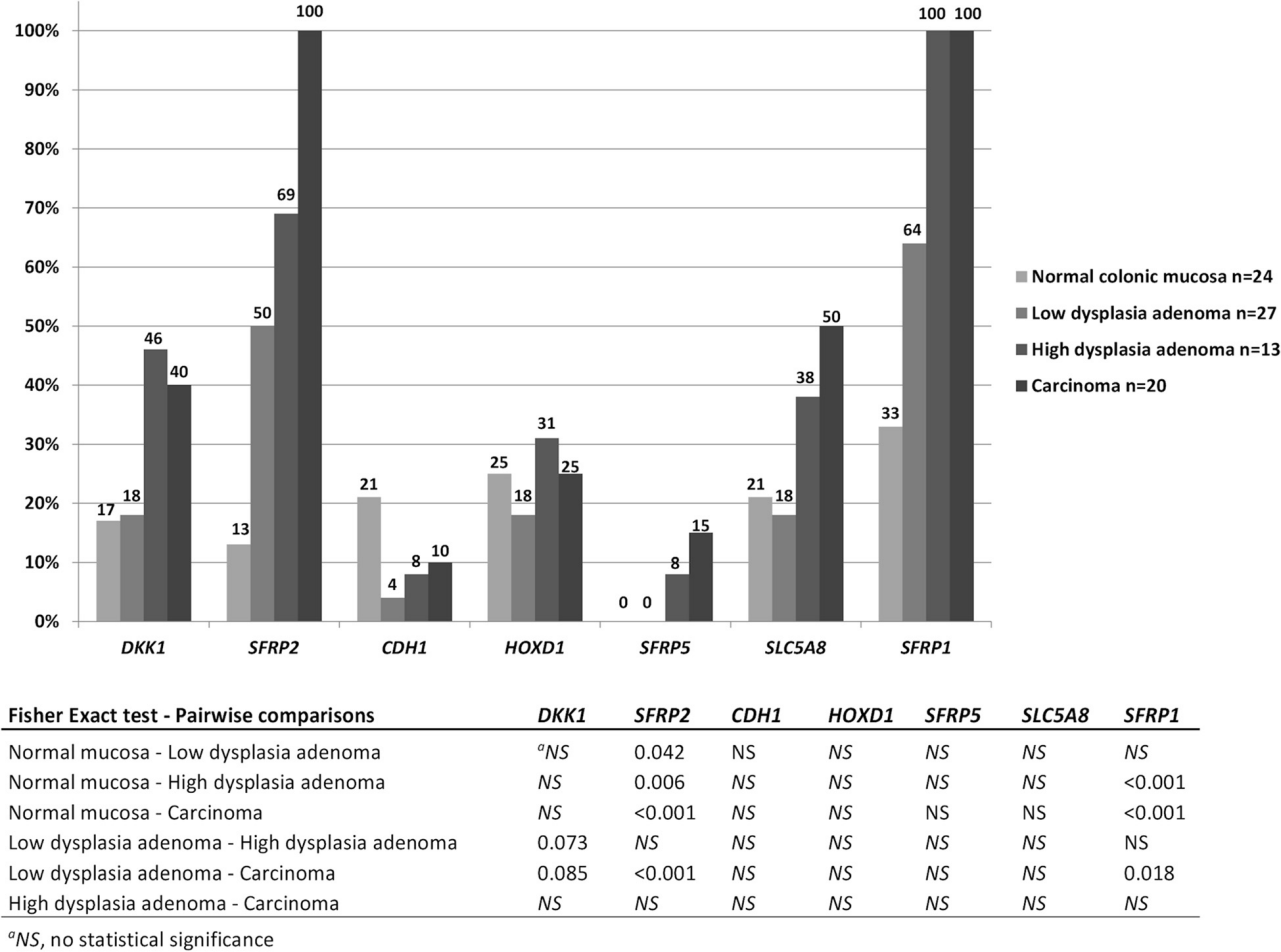
Frequency of hypermethylated candidate genes in the LS retrospective series. Numerical values of percentages are given above each bar. Hypermethylation thresholds were calculated according to stringency level I. Pairwise comparisons were calculated by Fisher’s exact test (two-sided *p* values), and the *p* values were adjusted for multiple comparisons by Bonferroni correction

In the retrospective LS series, hypermethylation frequencies for *SFRP2* were significantly higher in all tumor types when compared to normal mucosa (Fig. 3). Importantly, this included adenomas with low-grade dysplasia already (50 % vs. 13 %, *p* = 0.042). For *SFRP1*, significantly increased frequencies of hypermethylation were only observed in adenomas with high-grade dysplasia and carcinomas (Fig. 3).

### Correlation of candidate gene methylation with mRNA expression in cancer cell lines

To evaluate functional significance of promoter methylation, MMR-deficient colorectal, endometrial, and ovarian cancer cell lines (Additional file 5: Table S4) were treated with the demethylating agent 5-aza-CdR and the histone deacetylase inhibitor TSA, followed by RNA profiling on microarrays. Consistent (1.8–7.2-fold) treatment-induced upregulation of *SFRP1* was seen in HCT15, HCT116, and HEC59 analogous to LS-associated CRC and endometrial cancer. *SFRP2* was significantly upregulated (1.9-fold) in HCT116. Upregulation was accompanied by reduced promoter methylation by MS-MLPA.

When methylation (Dm) values were plotted against mRNA expression in all studied cancer cell lines (treated and untreated) and the corresponding normal tissues, a significant inverse correlation was observed between *SFRP1* (*r* = −0.688, *p* = 0.001) and *SFRP2* (*r* = −0.657, *p* = 0.002) mRNA expression and methylation (Additional file 6: Figure S2). Moreover, *SFRP1* and *SFRP2* expressions were significantly lower in the untreated cancer cell lines compared to the corresponding normal controls (data not shown). Our data suggest that DNA methylation plays a significant role in the expressional regulation of these genes.

### Hypermethylation of CIMP and candidate genes vs. expressional status of MMR proteins

Eleven adenomas with low-grade dysplasia retained MMR protein expression (Table 2) suggesting that the second hit to inactivate the responsible MMR gene had not yet occurred. DNA was available for eight such adenomas, and 3/8, 4/8, 6/8, and 2/8 revealed hypermethylation of *IGF2*, *NEUROG1*, *SFRP1*, and *SFRP2*, respectively. The hypermethylation frequencies were essentially comparable to those in low-grade dysplasia adenomas with silenced MMR protein (15/18, 4/18, 11/18, and 10/18 for the respective loci). The results suggest that hypermethylation of the investigated genes can occur prior to somatic inactivation of the predisposing MMR gene.

### Effect of age at biopsy on normal colonic tissue methylation

Since DNA methylation tends to increase with age [14], the level of methylation in each prospectively collected colorectal mucosa specimen was evaluated against the chronological age of the individual at the time of biopsy. A moderate-to-strong positive correlation was detected between age at biopsy and normal colonic mucosa Dm values corresponding to *IGF2* probes I (*r* = 0.694, *p* < 0.0001), II (*r* = 0.726, *p* < 0.0001), and III (*r* = 0.742, *p* < 0.0001) and *NEUROG1* probes I (*r* = 0.566, *p* < 0.0001), III (*r* = 0.703, *p* < 0.0001), and IV (*r* = 0.655, *p* < 0.0001) (Additional file 7: Figure 3SA–B). Additionally, moderate correlation between age at biopsy and *SFRP1* (*r* = 0.554, *p* < 0.0001), *SFRP2* (*r* = 0.550, *p* < 0.0001), and *SLC5A8* (*r* = 0.554, *p* < 0.0001) methylation was observed in normal colonic mucosa (Additional file 7: Figure S3C). This indicates that aging itself increases methylation of the CIMP markers in the histologically normal mucosa. No age-related correlation was observed for *MLH1* region C methylation.

### Analysis of field defects in histologically normal colonic mucosa

To investigate if aberrant DNA methylation might form carcinogenic “fields” in the histologically normal mucosa, colonic mucosa biopsies of the prospective LS series were evaluated for hypermethylation of CIMP markers and candidate genes. The individuals were divided into four groups depending on age at biopsy and absence vs. presence of (previous or concurrent) CRC (groups 1–4). The first two groups included mutation carriers 50 years old and below and consisted of 22 individuals without CRC (group 1) and 6 individuals with CRC (group 2). The remaining two groups included mutation carriers above 50 years and consisted of 17 individuals without CRC (group 3) and 10 individuals with CRC (group 4). The interval between CRC diagnosis and time of biopsy was 5.1 years (range 0–11.5) in group 2 and 5.5 years (range 0–12.4) in group 4. Average age at biopsy was comparable in group 1 (35, range 26–50) vs. group 2 (43, range 39–48) and in group 3 (61, range 51–75) vs. group 4 (63, range 51–74), excluding the age effect as a possible confounder in the respective comparisons.

When the effect of CRC on CIMP marker methylation was examined within the age groups (≤50 and above 50), no significant differences were observed (Additional file 8: Figure S4). However, age effect was evident from comparisons of the “under 50, no CRC” with the “over 50, no CRC” groups and “under 50, CRC” with the “over 50, CRC” groups, which revealed significant increases in methylation for several *IGF2* and *NEUROG1* probes (Additional file 8: Figure S4).

**Fig. 4:**
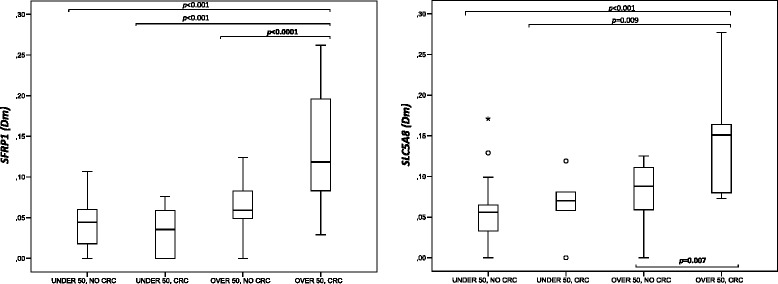
Effect of aging and previously diagnosed CRC on *SFRP1* and *SLC5A8* normal colonic mucosa Dm values. Study groups: patients ≤50 years without (*n* = 22) and with (*n* = 6) a CRC diagnosis, and patients >50 years without (*n* = 17) and with (*n* = 10) a CRC diagnosis. Statistical testing was performed with one-way ANOVA and Tukey’s post hoc test or Kruskal-Wallis one-way ANOVA

The candidate genes, too, showed an age effect, but despite it, significant increases in methylation were observed for *SFRP1* (*p* < 0.0001) and *SLC5A8* (*p* = 0.007) in the “over 50 group,” when stratified by the presence vs. absence of previously diagnosed CRC (Fig. 4). Moreover, our results from duplicate MS-MLPA assays (“Methods” section) as well as from dilution experiments (Additional file 9: Figure S5) showed that the observed changes in methylation were unlikely to be explained by technical variation or other similar reasons. Thus, our finding may indicate a potential field defect in normal mucosa.

## Discussion

Studies on sporadic CRC have demonstrated that promoter hypermethylation can act as an alternative mechanism to mutations, having a causal role in colorectal tumorigenesis [5, 3]. Promoter methylation can be detected in CRC-associated normal colonic mucosa and aberrant crypt foci (ACF), the earliest identifiable neoplastic lesions of the colon [15, 16]. Aging increases genomic DNA methylation [14] and is therefore particularly relevant to sporadic CRC which develops two to three decades later than hereditary CRC. We utilized unique features of LS, including increased tumor incidence and the availability of multiple specimens per individual (normal mucosa, adenoma, and carcinoma, taken at different time points), to gain insights into the role of DNA methylation alterations in hereditary CRC.

Loss of MMR protein corresponding to the predisposing MMR gene increased with the degree of dysplasia (Table 2), in agreement with results from a smaller series of screen-detected LS-adenomas [17]. In addition, we demonstrate that inactivation of *MLH1* by somatic methylation can constitute a “second hit” in LS-associated tumorigenesis. The method of methylation analysis (notably, the ability for quantification of methylation) and the particular region(s) of MMR gene promoters evaluated are important factors to take into account in “second hit” analyses. While one previous study [18] detected *MLH1* methylation in 10/18 (56 %) of LS-adenomas by methylation-specific PCR, which is not a quantitative method, our frequency (1/30, 3 %, for region C methylation in adenomas with silenced MLH1 protein) complies with other quantitative studies for the same region suggesting that in a small but definite proportion (2–15 %) of tumors from LS patients the wild-type allele is inactivated by *MLH1* promoter hypermethylation [19–22]. Hypermethylation of region A of *MLH1* was relatively common (up to 60 %) in the LS samples (Fig. 1). Methylation of region A correlates with age and does not silence gene expression, whereas methylation of region C (located closely upstream to the transcription start site) correlates with gene silencing [12, 21, 23]. Moreover, the single adenoma with *MLH1* promoter methylation in region C in our investigation was CIMP(+) by all three criteria tested, supporting previous evidence of MMR gene methylation as the “second hit” being associated with a more general CIMP in the tumors [19, 24].

The fact that almost half of the low-grade dysplasia adenomas retained MMR protein expression suggests that somatic inactivation of the wild-type allele may not always precede polyp formation, consistent with Yurgelun et al. [25]. In such cases, the existence of other somatic driver events has been hypothesized [26]. Moreover, non-tumorous mucosa from resections for intestinal cancer revealed a frequent occurrence of MMR-deficient crypt foci in LS mutation carriers, which contrasts with the low number of adenomas and carcinomas becoming clinically manifested and underlines the necessity of other tumorigenic events before or after MMR gene inactivation [27]. DNA methylation changes are excellent candidates for such events. The exact step at which the most abundant or important DNA methylation changes may occur remains unsettled. In the sporadic setting, a number of studies conclude that most alterations disrupting the normal patterns of DNA methylation occur in precursor lesions (ACF and polyps) rather than at more advanced stages (carcinoma and metastasis) [28]. On the other hand, Beggs et al. [7] detected the bulk of promoter hypermethylation in the transition from adenoma to carcinoma, rather than from normal tissue to adenoma. Ibrahim et al. [29] found that DNA methylation changes take place sequentially at specific transition points involving *RUNX3* in normal colonic mucosa, *NEUROG1* and *CACNA1G* in hyperplastic polyps, *SFRP2* and *IGF2*-DMR0 in adenomatous polyps, and *CDKN2A* and *MLH1* in the adenocarcinoma stage. We chose to use previously established CIMP markers and panels and found that CpG island methylation in LS adenomas and carcinomas increases with dysplasia (Figs. 1 and 2). Among the individual markers, the frequency of specimens with hypermethylation of *IGF2* and *NEUROG1* was significantly increased in adenomas with low-grade dysplasia already (Fig. 1), including some with the predisposing MMR gene not silenced, yet, indicating that methylation of *IGF2* and *NEUROG1* may provide a marker of early colon oncogenesis.

In sporadic colorectal tumors, MMR deficiency is mainly proposed to serve cancer progression rather than initiation [30]. Moreover, analysis of microdissected glands revealed significant heterogeneity for MSI and promoter methylation of MMR genes within individual polyps [31]. Our IHC and MSI results (Table 2), like those by Yurgelun et al. [25], suggest the possibility that some LS adenomas might be initiated by mechanisms other than MMR deficiency and that the MMR and non-MMR pathways may converge in later stages (adenomas with high-grade dysplasia and carcinomas). CIMP and candidate genes were often hypermethylated in low-grade dysplasia adenomas from our series regardless of MMR status of the tumors; thus, aberrant methylation might be among the earliest events in colorectal tumors developing in MMR gene mutation carriers.

Apart from adenomatous polyps that are viewed as precursors of CRC, LS mutation carriers may also exhibit polyps that develop along the serrated pathway, including hyperplastic polyps [32]. Hyperplastic polyps are traditionally considered to be non-neoplastic, lacking potential for malignant progression [33]. The latter view is supported by observations of a virtual absence of MMR defects in hyperplastic polyps from LS mutation carriers [25, 34]. A gland-level analysis of hyperplastic polyps from sporadic cases has shown frequent MSI and methylation aberrations, and the possibility that such polyps might be precursors to MSI CRC cannot be ruled out [31]. In light of the findings described above, the frequent hypermethylation we observed for the markers from the CIMP panel (Additional file 3: Figure S1A) and candidate gene panel (Additional file 3: Figure S1B) in hyperplastic polyps from LS mutation carriers is interesting and warrants further studies to evaluate the significance of such methylation events in tumorigenesis.

Specific dietary compounds are known to act as important modifiers of the methylation patterns of the colon, and DNA methylation of the intestinal mucosa can thus link nutrition to cancer [35]. Here, we report significantly increased hypermethylation frequencies for two secreted frizzled-related proteins, *SFRP1* and *SFRP2,* in LS-associated adenomas and carcinomas when compared to normal colon (Fig. 3). These genes emerged from our previous dietary intervention study on the *Mlh1* mouse model [11]. In the normal colon epithelial cells, SFRPs function as Wnt signaling antagonists and compete with Wnt proteins for binding to their receptor, frizzled. Transcriptional silencing of these genes through promoter hypermethylation (epigenetic gatekeeper) activates the APC protein complex which further promotes cell proliferation and ACF formation [36]. Interestingly, hypermethylation of these genes with concomitant reduction in gene expression was reported to apply to both CIMP-high and non-CIMP tumors, which indicates that aberrant methylation of these genes may occur in colorectal tumors irrespective of their subtype [37].

Field defects are clonal abnormalities in the epithelial gene expression that precede cancer development and predispose to it, occasionally causing the simultaneous occurrence of multiple tumors within a field [38]. Age-related DNA methylation changes have been proposed as potential sources of field defects in the colon [16, 14]. Observations that DNA methylation in normal mucosa may associate with pathway-specific susceptibility to CRC [39, 40] and that synchronous cancer pairs share epigenetic features such as CIMP and LINE-1 methylation status [41] support the existence of epigenetic field defects in CRC development. In our investigation, mutation carriers over 50 years old with a previous CRC diagnosis showed a significantly elevated average degree of methylation of *SFRP1* and *SLC5A8* vs. cancer-free individuals of a comparable age. Differences between the two groups of mucosa were small though statistically significant. *SFRP1* was discussed above; *SLC5A8* encodes a sodium transporter that directly influences the absorption of short-chain fatty acids (e.g., butyrate, a histone deacetylase inhibitor) from the apical membrane of the intestinal tract into the colon [42] and is frequently silenced in ACF and CRC by promoter methylation [43]. Our findings are consistent with a recent epigenome-wide study by Luo et al. who identified 65 loci with higher methylation in (sporadic) CRC-associated mucosa vs. colon mucosa from cancer-free individuals [44].

## Conclusions

We show that increased DNA methylation of CIMP markers and candidate gene loci accompanies tumor progression in LS. Methylation alterations may form carcinogenic fields in histologically normal mucosa and occur in adenomas at a stage where MMR protein expression is still intact. When affecting the predisposing MMR gene, promoter methylation can constitute the somatic “second hit”. Our results provide new insights into the multistep colorectal tumorigenesis in LS and CRC in general [29, 36, 43]. The findings also pinpoint potential markers for early detection and the assessment of progression of CRC. Altered methylation at such marker loci may help identify individuals prone to develop CRC through CIMP who might benefit from demethylating agents for chemoprevention or treatment [45].

## Methods

### Patient samples

CRC families represented the nationwide Hereditary Colorectal Cancer Registry of Finland. Colorectal fresh frozen biopsies (prospective series, Table 1) were gathered from LS and FAP patients during colonoscopy screenings and colectomies performed at the Helsinki University Central Hospital and Jyväskylä Central Hospital during 10/2011–5/2013. Normal mucosa biopsies were collected from one to four distinct colonic regions. In addition, blood was drawn to provide another source of normal cells for comparison. Forty-three individuals from the prospective series also contributed archival specimens (retrospective series, Table 1; predisposing MMR gene mutations specified in Additional file 10: Table S5). The histology of adenomas and carcinomas was verified by one of the authors (A.R). Carcinoma sections for DNA extractions contained 30–80 % tumor epithelium (average 50 %). The Institutional Ethics Board of Central Finland Health Care District approved the collection of biopsies during surveillance (K-S shp Dnro 10U/2011). The National Authority for Medicolegal Affairs (Dnro 1272/04/044/07) approved the collection of archival specimens.

### Immunohistochemistry (IHC) for MMR protein expression

IHC was performed by standard procedures [46] with the following primary antibodies: MLH1 (clone ES05; 75 mg/l; Dako North America, Inc. CA), MSH2 (clone G219-1129; 0.5 mg/ml; BD Pharmingen), MSH6 (clone EP49, AC00-47, Epitomics, Burlingame, CA), and PMS2 (clone EPR3947, 0.324 mg/ml, Abcam, Cambridge, UK). Negative cancer cell immunostaining was interpreted to indicate inactivation of the respective MMR gene.

### Microsatellite instability (MSI) analysis

MSI analysis was based on the mononucleotide repeat markers *BAT25* and *BAT26*, which are sensitive and specific indicators of the MSI-high phenotype [47, 48]. Tumors with unstable *BAT25* or *BAT26* were considered to have MSI, whereas those with normal *BAT25* and *BAT26* were microsatellite-stable.

### Methylation-specific multiplex ligation-dependent probe amplification (MS-MLPA) for methylation analysis

MS-MLPA probes contain a recognition sequence (GCGC) for the methylation-sensitive endonuclease HhaI, and methylated template DNA generates a signal peak [49]. MS-MLPA was performed according to the manufacturer’s instructions [50]. The methylation dosage ratios (Dm) were calculated separately for each normal mucosa and tumor sample (Additional file 1: Table S1) as described [51]. The Dm value of 0.15 or above (corresponding to 15 % of methylated DNA) was treated as the conservative technical threshold for methylation detection [49]. Since the baseline level for methylation in normal tissue may vary between probes (Additional file 1: Table S1), normal mucosa specimens were used to determine thresholds for hypermethylation in tumor tissues (Additional file 2: Table S2). Dm values in each tumor were compared to these thresholds to determine whether or not the tumor was hypermethylated at the respective locus. The results from individual tumors were then combined to calculate the hypermethylation frequency for each tumor type. The hypermethylation threshold (separate for fresh frozen and archival tissue-derived DNA) was defined as the mean Dm in normal mucosa plus 1 standard deviation (stringency level I) or 2 standard deviations (stringency level II) with the purpose to achieve an optimal discrimination between normal and tumor tissues. Stringency level I was used in all contexts except for classification of tissue specimens into CIMP(+) and CIMP(−) categories where stringency level II provided a better distinction (Fig. 2). In the prospective series, an average Dm value incorporating 1–4 colorectal regions was calculated to describe the normal mucosa Dm of each individual.

MS-MLPA was found to be highly reproducible (an average difference of Dm = 0.029 was observed between 288 replicate measurements) and sensitive (even 5 % methylation could be reliably detected in most cases; Additional file 9: Figure S5).

#### MMR gene promoter methylation

For studies of the “second hit,” the methylation status of MMR genes was analyzed by SALSA MLPA probemix ME011 (MRC Holland, Amsterdam, The Netherlands). Analyzed *MLH1* promoter regions corresponded to regions A, B, and C as described by Deng et al. [12].

#### CpG island methylator phenotype (CIMP)

Promoter methylation of *CACNA1G*, *IGF2*, *NEUROG1*, *RUNX3*, *SOCS1*, *CDKN2A*, *MLH1*, and *CRABP1* was studied by SALSA MLPA probemix ME042 (MRC Holland, Amsterdam, The Netherlands). The probemix contains 3–6 probes for each CIMP marker gene. A gene was considered methylated when one fourth (25 %) or more probes were methylated [13]. Two alternative marker panels were utilized to classify colorectal tumors as CIMP(+) or CIMP(−). The Weisenberger panel [52, 53] includes five genes (*CACNA1G*, *SOCS1*, *RUNX3*, *IGF2*, and *NEUROG1*), at least three of which should be methylated for CIMP(+) [13]. The Ogino panel includes three additional genes (*CDKN2A*, *MLH1*, and *CRABP1*) [52, 53], and five or more methylated genes out of eight (Ogino 5/8, CIMP-low) or six or more methylated genes out of eigth (Ogino 6/8, CIMP-high) were regarded to indicate CIMP(+).

#### Custom MS-MLPA for methylation analysis of candidate genes

To study promoter methylation of *DKK1*, *SFRP1*, *SFRP2*, *SFRP5*, *CDH1*, *HOXD1*, and *SLC5A8* [11], a custom MS-MLPA assay was designed. CpG islands (CGI) were identified by EMBOSS CpGplot [54] and CpG Island Searcher [55]. DNAs from cancer cell lines and normal tissues were bisulfite sequenced (with primers specified in Additional file 4: Table S3A) to determine the methylation statuses of the CpG sites within the CGIs. Custom MS-MLPA probes were designed to target GCGC sites (Additional file 4: Table S3B). Salsa MLPA P300-A2 Human DNA Reference-2 (MRC Holland, Amsterdam, The Netherlands) was added to the custom designed MS-MLPA probe mix. The custom assay was optimized against bisulfite sequencing as described [56], resulting in the conservative technical threshold of Dm ≥ 0.15 for methylation detection.

### Epigenetic drug treatments and analysis of genome-wide mRNA expression

Cancer cell lines (Additional file 5: Table S4) were treated with epigenetic drugs and RNA expression was profiled on Affymetrix Human Genome U133 Plus 2.0 GeneChip® microarrays (Affymetrix, Santa Clara, CA) as described [56]. Normal tissue DNA and RNA were purchased from Amsbio (Abingdon, UK). Microarray data were analyzed by GeneSpring GX software, version 12 (Agilent Technologies, Santa Clara, CA) using RMA normalization. Statistically significant gene expression changes were identified by moderated *t* test combined with the Benjamini and Hochberg correction for multiple testing and by using filters based on *p* value cutoff of 0.05 and fold change cutoff of +/−1.5. The mRNA expression profiling data have been submitted to GEO (accession number: GSE58058).

### Statistical analysis

Statistical analysis of methylation data was performed using the SPSS software, version 20.0 (IBM SPSS Inc. Chicago, IL, USA). Frequency of methylated target sites in each type of tissue specimen was calculated separately for each gene using the probe-specific threshold values. Two-sided *p* values were calculated for each pairwise comparison by Fisher’s exact test and adjusted for multiple comparisons by Bonferroni correction. Pearson product-moment correlation coefficient (*r*) was used to study the correlation of methylation and expression and the association between age at biopsy and methylation in normal colonic mucosa (individual average normal colon Dm values). Statistical significance of methylation differences between groups studied for field defects was tested by one-way ANOVA, and Tukey’s post hoc test was used for pairwise comparisons. Alternatively, the non-parametric test Kruskal-Wallis one-way ANOVA (*k* samples, pairwise comparisons) was utilized for series not normally distributed. Homogeneity of variances was tested by Levene’s test and normality by Shapiro-Wilk test. *P* values <0.05 were considered significant.

## Additional files

Additional file 1: Table S1. Average methylation dosage ratios (Dm) and standard deviations for normal colonic mucosa and each tumor type. Additional file 2: Table S2. Threshold values for hypermethylation at each gene locus in the Lynch syndrome retrospective series calculated based on normal colonic mucosa specimens. Additional file 3: Figure S1. Frequency of hypermethylated samples in the LS and FAP prospective series. Additional file 4: Table S3. (A) Bisulfite primer sequences and (B) MS-MLPA probe design for candidate gene panel. Additional file 5: Table S4. Cell line characteristics. Additional file 6: Figure S2. Correlation between *SFRP1* and *SFRP2* mRNA expression and methylation. Additional file 7: Figure S3. Correlation of age and normal colonic mucosa methylation. (A) *IGF2* probes I, II, and III. (B) *NEUROG1* probes I, III, and IV. (C) *SFRP1*, *SFRP2*, and *SLC5A8*. Additional file 8: Figure S4. Effect of aging and previously diagnosed CRC on normal colonic mucosa methylation. (A) *IGF2* (probes I–III). (B) *NEUROG1* (probes I, III, IV, and VI). Additional file 9: Figure S5. MS-MLPA sensitivity for (A) CIMP and (B) candidate gene panel. Results obtained with a gradient of decreasing amount of methylated control DNA diluted into a solution of unmethylated control DNA indicate that even 5 % of methylated DNA can be reliably detected. Additional file 10: Table S5. Germline mutation specifications for patients (retrospective series).

## Abbreviations

5-AZA-CdR: 5-aza-2′ deoxycytidine
ACF: aberrant crypt foci
CGI: CpG island
CIMP: CpG island methylator phenotype
CRC: colorectal cancer
FAP: familial adenomatous polyposis
FFPE: formalin-fixed paraffin-embedded
LINE: long interspersed element
LS: Lynch syndrome
MMR: mismatch repair
MSI: microsatellite instability
MS-MLPA: methylation-specific multiplex ligation-dependent probe amplification
MSS: microsatellite stable
SFRP: secreted frizzled-related protein
TSA: trichostatin A

## References

[1] Vasen HF, Blanco I, Aktan-Collan K, Gopie JP, Alonso A, Aretz S (2013). Revised guidelines for the clinical management of Lynch syndrome (HNPCC): recommendations by a group of European experts. Gut.

[2] Geiersbach KB, Samowitz WS (2011). Microsatellite instability and colorectal cancer. Arch Pathol Lab Med.

[3] Kane MF, Loda M, Gaida GM, Lipman J, Mishra R, Goldman H (1997). Methylation of the hMLH1 promoter correlates with lack of expression of hMLH1 in sporadic colon tumors and mismatch repair-defective human tumor cell lines. Cancer Res.

[4] Veigl ML, Kasturi L, Olechnowicz J, Ma AH, Lutterbaugh JD, Periyasamy S (1998). Biallelic inactivation of hMLH1 by epigenetic gene silencing, a novel mechanism causing human MSI cancers. Proc Natl Acad Sci U S A.

[5] Lao VV, Grady WM (2011). Epigenetics and colorectal cancer. Nat Rev Gastroenterol Hepatol.

[6] Kim YH, Petko Z, Dzieciatkowski S, Lin L, Ghiassi M, Stain S (2006). CpG island methylation of genes accumulates during the adenoma progression step of the multistep pathogenesis of colorectal cancer. Genes, Chromosomes Cancer.

[7] Beggs AD, Jones A, El-Bahrawy M, Abulafi M, Hodgson SV, Tomlinson IP (2013). Whole-genome methylation analysis of benign and malignant colorectal tumours. J Pathol.

[8] Nosho K, Irahara N, Shima K, Kure S, Kirkner GJ, Schernhammer ES (2008). Comprehensive biostatistical analysis of CpG island methylator phenotype in colorectal cancer using a large population-based sample. PLoS One.

[9] McCabe MT, Brandes JC, Vertino PM (2009). Cancer DNA methylation: molecular mechanisms and clinical implications. Clinical cancer research: an official journal of the American Association for Cancer Research.

[10] Mecklin JP, Aarnio M, Laara E, Kairaluoma MV, Pylvanainen K, Peltomaki P (2007). Development of colorectal tumors in colonoscopic surveillance in Lynch syndrome. Gastroenterology.

[11] Pussila M, Sarantaus L, Dermadi Bebek D, Valo S, Reyhani N, Ollila S (2013). Cancer-predicting gene expression changes in colonic mucosa of Western diet fed Mlh1+/− mice. PLoS One.

[12] Deng G, Chen A, Hong J, Chae HS, Kim YS (1999). Methylation of CpG in a small region of the hMLH1 promoter invariably correlates with the absence of gene expression. Cancer Res.

[13] Berg M, Hagland HR, Soreide K (2014). Comparison of CpG island methylator phenotype (CIMP) frequency in colon cancer using different probe- and gene-specific scoring alternatives on recommended multi-gene panels. PLoS One.

[14] Issa JP (2000). CpG-island methylation in aging and cancer. Curr Top Microbiol Immunol.

[15] Chan AO, Broaddus RR, Houlihan PS, Issa JP, Hamilton SR, Rashid A (2002). CpG island methylation in aberrant crypt foci of the colorectum. Am J Pathol.

[16] Shen L, Kondo Y, Rosner GL, Xiao L, Hernandez NS, Vilaythong J (2005). MGMT promoter methylation and field defect in sporadic colorectal cancer. J Natl Cancer Inst.

[17] De Jong AE, Morreau H, Van Puijenbroek M, Eilers PH, Wijnen J, Nagengast FM (2004). The role of mismatch repair gene defects in the development of adenomas in patients with HNPCC. Gastroenterology.

[18] Kaz A, Kim YH, Dzieciatkowski S, Lynch H, Watson P, Kay Washington M (2007). Evidence for the role of aberrant DNA methylation in the pathogenesis of Lynch syndrome adenomas. International journal of cancer Journal international du cancer.

[19] Ollikainen M, Hannelius U, Lindgren CM, Abdel-Rahman WM, Kere J, Peltomaki P (2007). Mechanisms of inactivation of MLH1 in hereditary nonpolyposis colorectal carcinoma: a novel approach. Oncogene.

[20] Rahner N, Friedrichs N, Steinke V, Aretz S, Friedl W, Buettner R (2008). Coexisting somatic promoter hypermethylation and pathogenic MLH1 germline mutation in Lynch syndrome. J Pathol.

[21] Parsons MT, Buchanan DD, Thompson B, Young JP, Spurdle AB (2012). Correlation of tumour BRAF mutations and MLH1 methylation with germline mismatch repair (MMR) gene mutation status: a literature review assessing utility of tumour features for MMR variant classification. J Med Genet.

[22] Moreira L, Munoz J, Cuatrecasas M, Quintanilla I, Leoz ML, Carballal S (2015). Prevalence of somatic mutl homolog 1 promoter hypermethylation in Lynch syndrome colorectal cancer. Cancer.

[23] Nakagawa H, Chadwick RB, Peltomaki P, Plass C, Nakamura Y, de La Chapelle A (2001). Loss of imprinting of the insulin-like growth factor II gene occurs by biallelic methylation in a core region of H19-associated CTCF-binding sites in colorectal cancer. Proc Natl Acad Sci U S A.

[24] Nagasaka T, Rhees J, Kloor M, Gebert J, Naomoto Y, Boland CR (2010). Somatic hypermethylation of MSH2 is a frequent event in Lynch syndrome colorectal cancers. Cancer Res.

[25] Yurgelun MB, Goel A, Hornick JL, Sen A, Turgeon DK, Ruffin MT (2012). Microsatellite instability and DNA mismatch repair protein deficiency in Lynch syndrome colorectal polyps. Cancer prevention research.

[26] Lynch HT, Snyder CL, Shaw TG, Heinen CD, Hitchins MP (2015). Milestones of Lynch syndrome: 1895–2015. Nat Rev Cancer.

[27] Kloor M, Huth C, Voigt AY, Benner A, Schirmacher P, von Knebel Doeberitz M (2012). Prevalence of mismatch repair-deficient crypt foci in Lynch syndrome: a pathological study. Lancet Oncol.

[28] Carmona FJ, Esteller M (2010). Epigenomics of human colon cancer. Mutat Res.

[29] Ibrahim AE, Arends MJ, Silva AL, Wyllie AH, Greger L, Ito Y (2011). Sequential DNA methylation changes are associated with DNMT3B overexpression in colorectal neoplastic progression. Gut.

[30] Homfray TF, Cottrell SE, Ilyas M, Rowan A, Talbot IC, Bodmer WF (1998). Defects in mismatch repair occur after APC mutations in the pathogenesis of sporadic colorectal tumours. Hum Mutat.

[31] Beggs AD, Domingo E, Abulafi M, Hodgson SV, Tomlinson IP (2013). A study of genomic instability in early preneoplastic colonic lesions. Oncogene.

[32] Burt RW (2015). Colonic polyps in Lynch syndrome. Dis Colon Rectum.

[33] Noffsinger AE, Hart J (2010). Serrated adenoma: a distinct form of non-polypoid colorectal neoplasia?. Gastrointest Endosc Clin N Am.

[34] Rijcken FE, van der Sluis T, Hollema H, Kleibeuker JH (2003). Hyperplastic polyps in hereditary nonpolyposis colorectal cancer. Am J Gastroenterol.

[35] Arasaradnam RP, Commane DM, Bradburn D, Mathers JC (2008). A review of dietary factors and its influence on DNA methylation in colorectal carcinogenesis. Epigenetics: official journal of the DNA Methylation Society.

[36] Suzuki H, Watkins DN, Jair KW, Schuebel KE, Markowitz SD, Chen WD (2004). Epigenetic inactivation of SFRP genes allows constitutive WNT signaling in colorectal cancer. Nat Genet.

[37] Hinoue T, Weisenberger DJ, Lange CP, Shen H, Byun HM, Van Den Berg D (2012). Genome-scale analysis of aberrant DNA methylation in colorectal cancer. Genome Res.

[38] Braakhuis BJ, Tabor MP, Kummer JA, Leemans CR, Brakenhoff RH (2003). A genetic explanation of Slaughter’s concept of field cancerization: evidence and clinical implications. Cancer Res.

[39] Worthley DL, Whitehall VL, Buttenshaw RL, Irahara N, Greco SA, Ramsnes I (2010). DNA methylation within the normal colorectal mucosa is associated with pathway-specific predisposition to cancer. Oncogene.

[40] Svrcek M, Buhard O, Colas C, Coulet F, Dumont S, Massaoudi I (2010). Methylation tolerance due to an O6-methylguanine DNA methyltransferase (MGMT) field defect in the colonic mucosa: an initiating step in the development of mismatch repair-deficient colorectal cancers. Gut.

[41] Nosho K, Kure S, Irahara N, Shima K, Baba Y, Spiegelman D (2009). A prospective cohort study shows unique epigenetic, genetic, and prognostic features of synchronous colorectal cancers. Gastroenterology.

[42] Thangaraju M, Cresci G, Itagaki S, Mellinger J, Browning DD, Berger FG (2008). Sodium-coupled transport of the short chain fatty acid butyrate by SLC5A8 and its relevance to colon cancer. Journal of gastrointestinal surgery: official journal of the Society for Surgery of the Alimentary Tract.

[43] Li H, Myeroff L, Smiraglia D, Romero MF, Pretlow TP, Kasturi L (2003). SLC5A8, a sodium transporter, is a tumor suppressor gene silenced by methylation in human colon aberrant crypt foci and cancers. Proc Natl Acad Sci U S A.

[44] Luo Y, Wong CJ, Kaz AM, Dzieciatkowski S, Carter KT, Morris SM (2014). Differences in DNA methylation signatures reveal multiple pathways of progression from adenoma to colorectal cancer. Gastroenterology.

[45] Kodach LL, Jacobs RJ, Voorneveld PW, Wildenberg ME, Verspaget HW, van Wezel T (2011). Statins augment the chemosensitivity of colorectal cancer cells inducing epigenetic reprogramming and reducing colorectal cancer cell ‘stemness’ via the bone morphogenetic protein pathway. Gut.

[46] Thiel A, Heinonen M, Kantonen J, Gylling A, Lahtinen L, Korhonen M (2013). BRAF mutation in sporadic colorectal cancer and Lynch syndrome. Virchows Archiv: an international journal of pathology.

[47] Loukola A, Eklin K, Laiho P, Salovaara R, Kristo P, Jarvinen H (2001). Microsatellite marker analysis in screening for hereditary nonpolyposis colorectal cancer (HNPCC). Cancer Res.

[48] Esemuede I, Forslund A, Khan SA, Qin LX, Gimbel MI, Nash GM (2010). Improved testing for microsatellite instability in colorectal cancer using a simplified 3-marker assay. Ann Surg Oncol.

[49] Nygren AO, Ameziane N, Duarte HM, Vijzelaar RN, Waisfisz Q, Hess CJ (2005). Methylation-specific MLPA (MS-MLPA): simultaneous detection of CpG methylation and copy number changes of up to 40 sequences. Nucleic Acids Res.

[50] MRC Holland. http://mrc-holland.com. Accessed 5 March 2015.

[51] Pavicic W, Perkio E, Kaur S, Peltomaki P (2011). Altered methylation at microRNA-associated CpG islands in hereditary and sporadic carcinomas: a methylation-specific multiplex ligation-dependent probe amplification (MS-MLPA)-based approach. Mol Med.

[52] Weisenberger DJ, Siegmund KD, Campan M, Young J, Long TI, Faasse MA (2006). CpG island methylator phenotype underlies sporadic microsatellite instability and is tightly associated with BRAF mutation in colorectal cancer. Nat Genet.

[53] Ogino S, Kawasaki T, Kirkner GJ, Kraft P, Loda M, Fuchs CS (2007). Evaluation of markers for CpG island methylator phenotype (CIMP) in colorectal cancer by a large population-based sample. The Journal of molecular diagnostics: JMD.

[54] EMBOSS CpGplot. http://www.ebi.ac.uk/Tools/seqstats/emboss_cpgplot. Accessed 5 March 2015.

[55] CpG Island Searcher. http://cpgislands.usc.edu. Accessed 5 March 2015.

[56] Niskakoski A, Kaur S, Staff S, Renkonen-Sinisalo L, Lassus H, Jarvinen HJ (2014). Epigenetic analysis of sporadic and Lynch-associated ovarian cancers reveals histology-specific patterns of DNA methylation. Epigenetics: official journal of the DNA Methylation Society.

