# Supplementary material for: DNA hypermethylation appears early and shows increased frequency with dysplasia in Lynch syndrome-associated colorectal adenomas and carcinomas
Source: Clin Epigenetics. 2015 Jul 22;7(1):71. doi: 10.1186/s13148-015-0102-4 (PMC4511034; doi:10.1186/s13148-015-0102-4)

Suppl. Fig.1. Frequency of hypermethylated samples in the LS and FAP prospective series. Numerical values of percentages are given above each bar. Hypermethylation thresholds were calculated according to stringency level I, resulting in values below the technical threshold for all probes technical except *IGF2 I*, *IGF2 III*, *NEUROG1 I* and *NEUROG1 IV* that had calculated thresholds 0.44, 0.23, 0.20 and 0.16, respectively. Pairwise comparisons were calculated by Fisher's exact test (2-sided *p* values) and the p values were adjusted for multiple comparisons by Bonferroni correction. Significant increase ($p<0.05$) in the frequency of methylated specimen compared to normal colon tissue is indicated by *.

A. CIMP panel

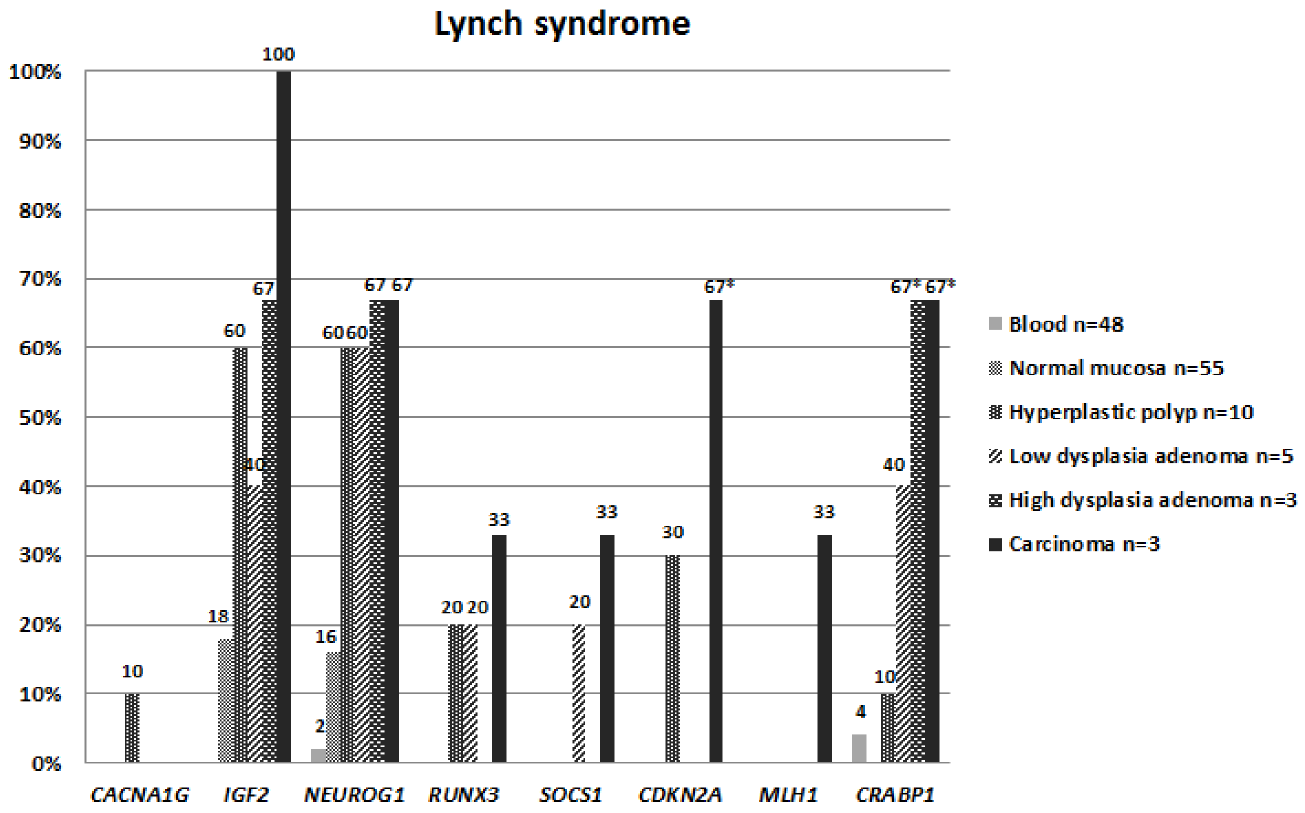

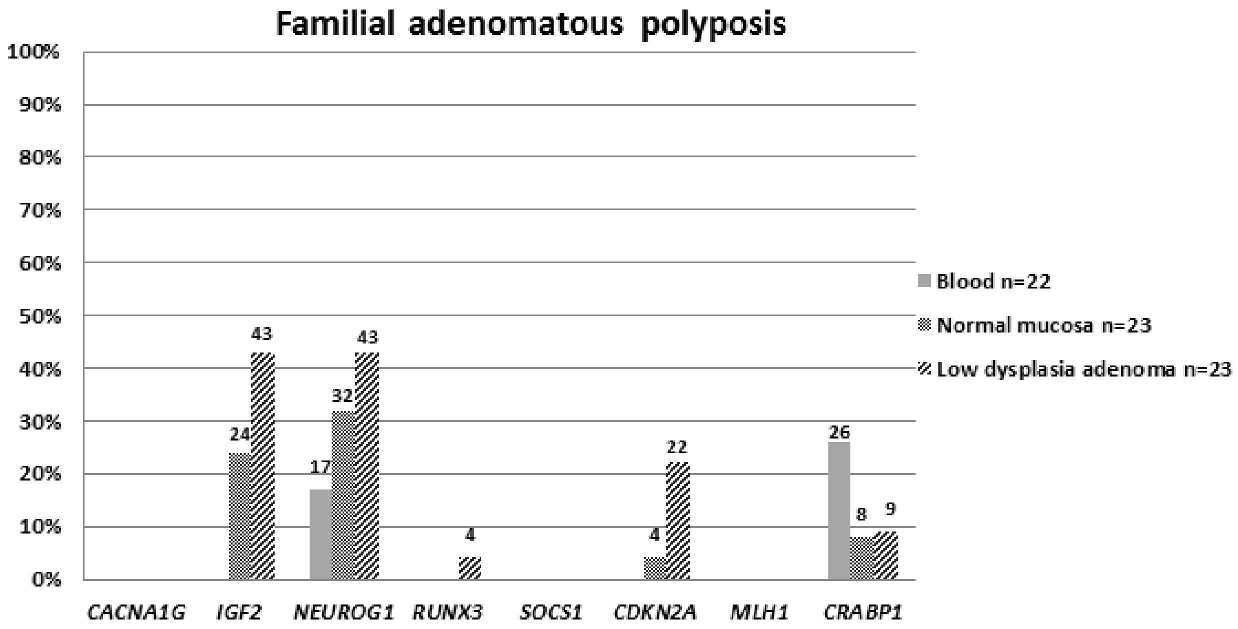

B. Candidate panel

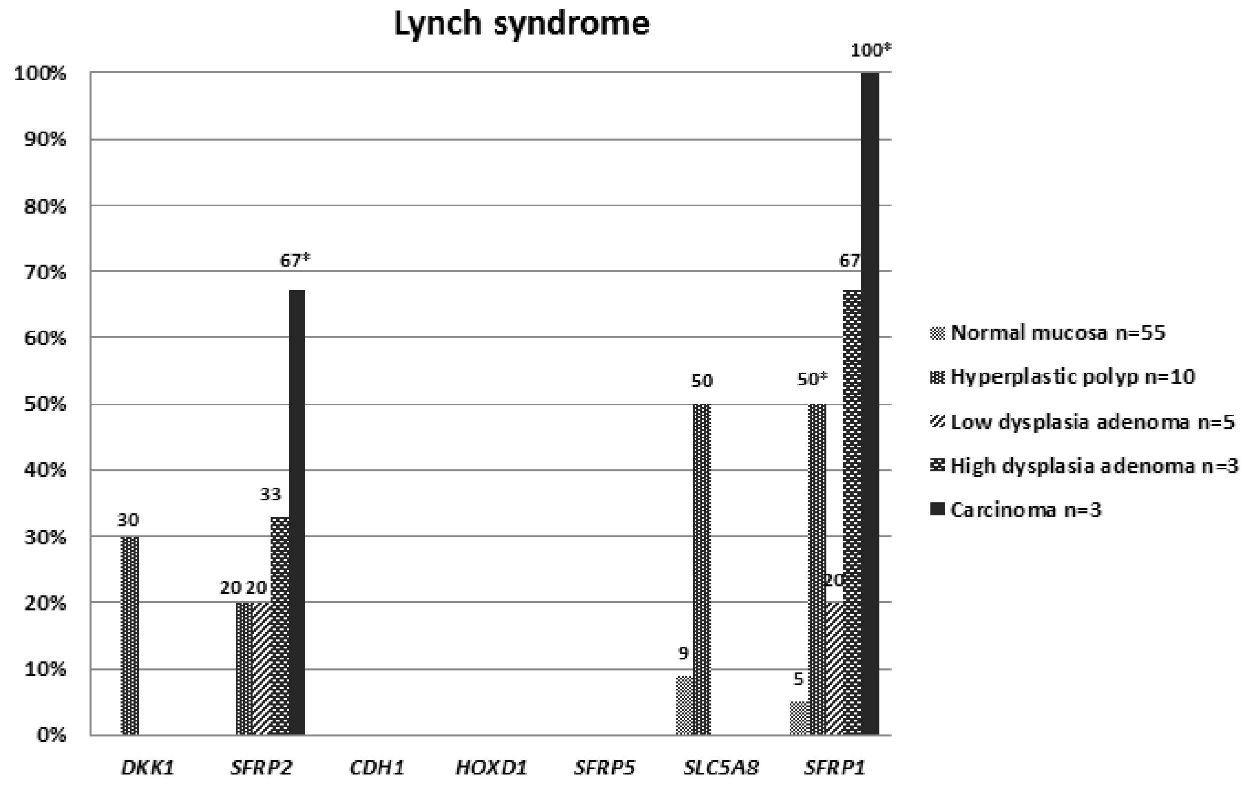

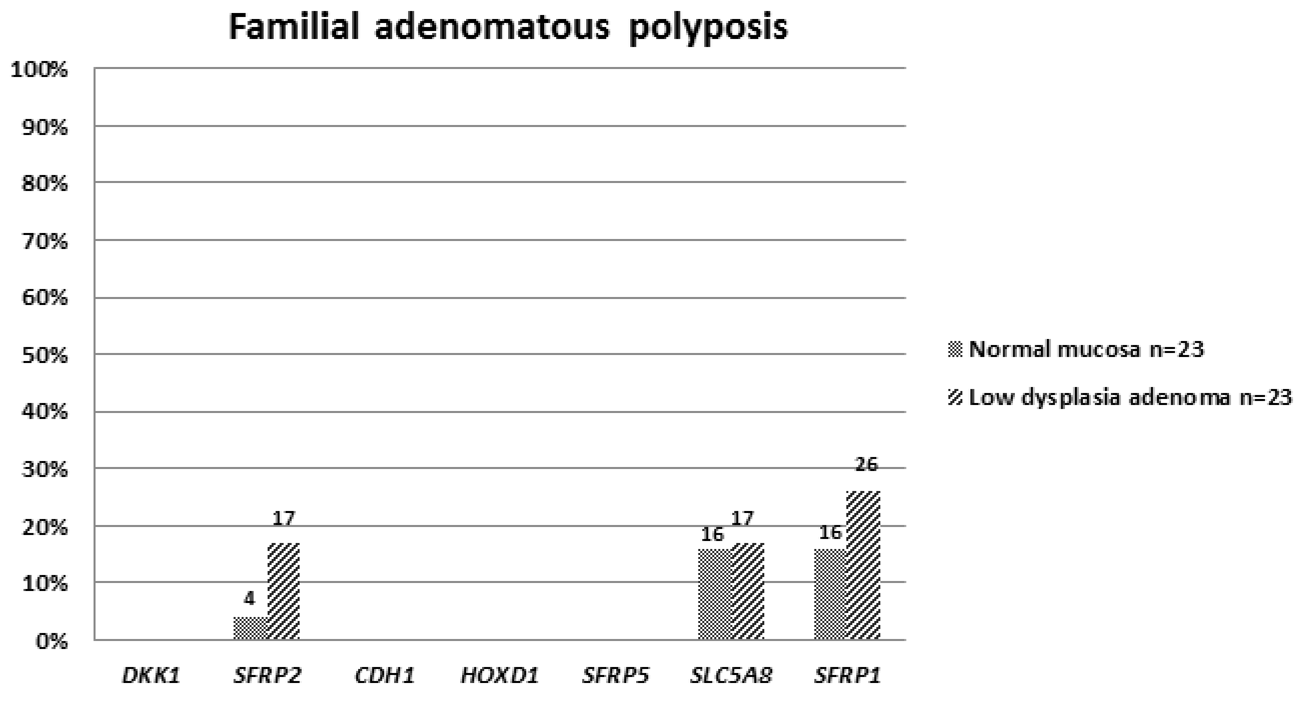

Supplement: Additional file 3: Figure S1. — Frequency of hypermethylated samples in the LS and FAP prospective series. [file 13148_2015_102_MOESM3_ESM.pdf]
