# Supplementary material for: DNA hypermethylation appears early and shows increased frequency with dysplasia in Lynch syndrome-associated colorectal adenomas and carcinomas
Source: Clin Epigenetics. 2015 Jul 22;7(1):71. doi: 10.1186/s13148-015-0102-4 (PMC4511034; doi:10.1186/s13148-015-0102-4)
Supplement: Additional file 4: Table S3. — (A) Bisulfite primer sequences and (B) MS-MLPA probe design for candidate gene panel. [file 13148_2015_102_MOESM4_ESM.pdf]

Suppl. Table 3. (A) Bisulfite primer sequences and (B) MS-MLPA probe design for candidate gene panel.

A.

Gene	Forward (5´→3´)	Reverse (5´→3´)	T_a	Size (bp)	Reference
DKK1	TTTTTTGAGTTTTTTTGAGATGATG	CTAACTACAAAACCTAAATACCCC	54°C/56°C	249	This study
SFRP2	AATTAGATTTAGAAAGTAGTGATTAGT	AACCAAAACCCTACAACATCRTAAAC	54°C	381	[1]
CDH1	ATTTATTTAGATTTTAGTAATTTTAGGTTAG	CAAATCAAAAAATCCRAAATACCTACAACA	50°C	393	This study
HOXD1	GTAGAGGATTTAGAAGAGGGGA	AAAAAAACAAAACTAAACRCTTAAATAAAAACC	52°C/54°C	221	[2] and this study
SFRP5	GGGAGGTAGGGAGTTTTGGGGAGAA	CCCAAATAAATAACAACCTACRCTAC	56°C	277	[1]
SLC5A8	GAGGTTTTATATTTGGGTTTGAGG	TAAACCCTACRCRCAAACTAATAACCC	54°C/56°C	222	[3] and this study
SFRP1	GTTTTGTTTTTTTAAGGGGTGTTGAG	ACACTAACTCCRAAAACTACAAAAC	54°C/56°C	209	[4]

B.

Gene	Probe length[a] (bp)	Left hybridizing sequence (LHS)[b]	Right hybridizing sequence (RHS)[b]	Distance of the HhaI site from ATG
DKK1	97	GGGAGTGA**GCGC**CACCTTGAACTCGGTT	CTCAATTCCAACGCTATCAAGAACCTG	+ 91 bp
SFRP2[c]	114	AACGGCTCATTCTGCTCCCCCGGGTCGGA	GCCCCCCGGAGCT**GCGC**GCGGGCTTGCA	- 198 bp
CDH1	119	CGACCGCACCCG**GCGC**CTGCCCTCGCT	CGGCGTCCCCGGCCAGCCATGGGCCCT	- 29 bp
HOXD1	124	CACCGGCC**GCGC**GTCACGCTCCCATTTAAC	CTTTTCCATGCCGCGACGCCCACACTCCGC	- 546 bp
SFRP5[c]	133	CCGAGCCGGGAGAGGG**GCGC**AAGACCT	G**GCGC**TGGGCGGGACGCTCGGGCAGGG	LHS - 333 bp & RHS - 318 bp
SLC5A8	139	CTGGAGGACGCCTCCAGTCCCCGCGGGAC	GCCACGCCT**GCGCGC**CAGGGATCCGGGAT	- 91 bp
SFRP1	158	GATTGGCT**GCGC**GGGGCGGCTCCGA	GGGC TCGGC CGTAGGAGCC CC GC	- 348 bp

[a] Each MS-MLPA probe pair consists of two separate hybridizing sequences (LHS and RHS).

[b] Hha1 restriction sites (GCGC) are in bold.

[c] Probe sequence published by Niskakoski et al. 2014 [5].

Refrerences

1. Cheng YY, Yu J, Wong YP, Man EP, To KF, Jin VX et al. Frequent epigenetic inactivation of secreted frizzled-related protein 2 (SFRP2) by promoter methylation in human gastric cancer. British journal of cancer. 2007;97(7):895-901. doi:10.1038/sj.bjc.6603968.
2. Jacinto FV, Ballestar E, Ropero S, Esteller M. Discovery of epigenetically silenced genes by methylated DNA immunoprecipitation in colon cancer cells. Cancer research. 2007;67(24):11481-6. doi:10.1158/0008-5472.CAN-07-2687.
3. Park JY, Helm JF, Zheng W, Ly QP, Hodul PJ, Centeno BA et al. Silencing of the candidate tumor suppressor gene solute carrier family 5 member 8 (SLC5A8) in human pancreatic cancer. Pancreas. 2008;36(4):e32-9. doi:10.1097/MPA.0b013e3181630ffe.
4. Meng Y, Wang QG, Wang JX, Zhu ST, Jiao Y, Li P et al. Epigenetic inactivation of the SFRP1 gene in esophageal squamous cell carcinoma. Digestive diseases and sciences. 2011;56(11):3195-203. doi:10.1007/s10620-011-1734-7.
5. Niskakoski A, Kaur S, Staff S, Renkonen-Sinisalo L, Lassus H, Jarvinen HJ et al. Epigenetic analysis of sporadic and Lynch-associated ovarian cancers reveals histology-specific patterns of DNA methylation. Epigenetics : official journal of the DNA Methylation Society. 2014;9(12):1577-87. doi:10.4161/15592294.2014.983374.
