# Supplementary material for: DNA hypermethylation appears early and shows increased frequency with dysplasia in Lynch syndrome-associated colorectal adenomas and carcinomas
Source: Clin Epigenetics. 2015 Jul 22;7(1):71. doi: 10.1186/s13148-015-0102-4 (PMC4511034; doi:10.1186/s13148-015-0102-4)
Supplement: Additional file 5: Table S4. — Cell line characteristics. [file 13148_2015_102_MOESM5_ESM.pdf]

Suppl. Table 4. Cell line characteristics.

**Cell line**	**Type of cancer**	**MMR Status**	**Reference**	**CIMP**	**Reference**
RKO	Colon carcinoma	Deficient, *MLH1* biallelic methylation	[1]	High	[2]
HCT15	Colon adenocarcinoma	Deficient, *MLH6* homozygous mutation	[3,4]	High	[2]
HCT116	Colorectal carcinoma	Deficient, *MLH1* hemizygous mutation	[5]	High	[2]
SKOV3	Ovarian serous adenocarcinoma	Deficient, Mutation in *MLH1*	[3,6]	High	[7]
ES2	Ovarian clear cell adenocarcinoma	Deficient	[1]	High	[7]
HEC59	Uterine endometrial carcinoma	Deficient, *MSH2* biallelic mutations	[3,8]	High	[2]
AN3CA	Uterine endometrial adenocarcinoma	Deficient, *MLH1* biallelic methylation	[1,9]	High	[2]

References

1. Veigl ML, Kasturi L, Olechnowicz J, Ma AH, Lutterbaugh JD, Periyasamy S et al. Biallelic inactivation of hMLH1 by epigenetic gene silencing, a novel mechanism causing human MSI cancers. Proceedings of the National Academy of Sciences of the United States of America. 1998;95(15):8698-702.
2. Joensuu EI, Abdel-Rahman WM, Ollikainen M, Ruosaari S, Knuutila S, Peltomaki P. Epigenetic signatures of familial cancer are characteristic of tumor type and family category. Cancer research. 2008;68(12):4597-605. doi:10.1158/0008-5472.CAN-07-6645.
3. Boyer JC, Umar A, Risinger JI, Lipford JR, Kane M, Yin S et al. Microsatellite instability, mismatch repair deficiency, and genetic defects in human cancer cell lines. Cancer research. 1995;55(24):6063-70.
4. Papadopoulos N, Nicolaides NC, Liu B, Parsons R, Lengauer C, Palombo F et al. Mutations of GTBP in genetically unstable cells. Science. 1995;268(5219):1915-7.
5. Papadopoulos N, Nicolaides NC, Wei YF, Ruben SM, Carter KC, Rosen CA et al. Mutation of a mutL homolog in hereditary colon cancer. Science. 1994;263(5153):1625-9.
6. Kim JH, Skates SJ, Uede T, Wong KK, Schorge JO, Feltmate CM et al. Osteopontin as a potential diagnostic biomarker for ovarian cancer. Jama. 2002;287(13):1671-9.
7. Niskakoski A, Kaur S, Staff S, Renkonen-Sinisalo L, Lassus H, Jarvinen HJ et al. Epigenetic analysis of sporadic and Lynch-associated ovarian cancers reveals histology-specific patterns of DNA methylation. Epigenetics : official journal of the DNA Methylation Society. 2014;9(12):1577-87. doi:10.4161/15592294.2014.983374.
8. Umar A, Koi M, Risinger JI, Glaab WE, Tindall KR, Kolodner RD et al. Correction of hypermutability, N-methyl-N'-nitro-N-nitrosoguanidine resistance, and defective DNA mismatch repair by introducing chromosome 2 into human tumor cells with mutations in MSH2 and MSH6. Cancer research. 1997;57(18):3949-55.
9. Kane MF, Loda M, Gaida GM, Lipman J, Mishra R, Goldman H et al. Methylation of the hMLH1 promoter correlates with lack of expression of hMLH1 in sporadic colon tumors and mismatch repair-defective human tumor cell lines. Cancer research. 1997;57(5):808-11.
