# Supplementary material for: DNA hypermethylation appears early and shows increased frequency with dysplasia in Lynch syndrome-associated colorectal adenomas and carcinomas
Source: Clin Epigenetics. 2015 Jul 22;7(1):71. doi: 10.1186/s13148-015-0102-4 (PMC4511034; doi:10.1186/s13148-015-0102-4)

Suppl. Fig. 2. Significant correlation between *SFRP1* (r= -0.688, p=0.001) and *SFRP2* (r= -0.657, p=0.002) mRNA expression and methylation. Tissue origin of cell lines is specified in Suppl. table 4. NC, normal colon; NE, normal endometrium; T, 5-aza-CdR and TSA treated cell line (without T, untreated).

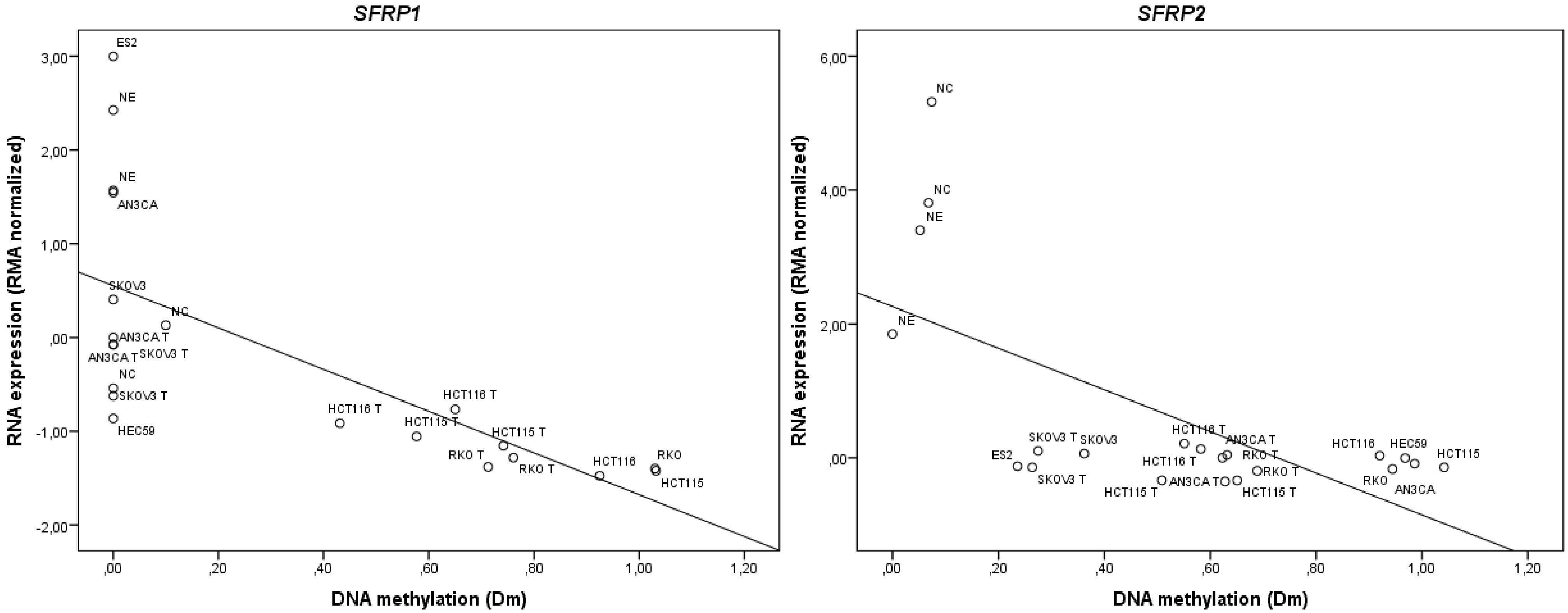

Supplement: Additional file 6: Figure S2. — Correlation between SFRP1 and SFRP2 mRNA expression and methylation. [file 13148_2015_102_MOESM6_ESM.pdf]
