# Supplementary material for: DNA hypermethylation appears early and shows increased frequency with dysplasia in Lynch syndrome-associated colorectal adenomas and carcinomas
Source: Clin Epigenetics. 2015 Jul 22;7(1):71. doi: 10.1186/s13148-015-0102-4 (PMC4511034; doi:10.1186/s13148-015-0102-4)
Supplement: Additional file 7: Figure S3. — Correlation of age and normal colonic mucosa methylation. (A) IGF2 probes I, II, and III. (B) NEUROG1 probes I, III, and IV. (C) SFRP1, SFRP2, and SLC5A8. [file 13148_2015_102_MOESM7_ESM.pdf]

Suppl. Fig. 3. Correlation of age and normal colonic mucosa methylation. (A) Strong correlation between age at biopsy and normal mucosa *IGF2* probes I ($r=0.694$, $p<0.0001$), II ($r=0.726$, $p<0.0001$) and III ($r=0.742$, $p<0.0001$) Dm values. **(B)** Strong correlation between age at biopsy and normal mucosa *NEUROG1* probes I ($r=0.566$, $p<0.0001$), III ($r=0.703$, $p<0.0001$) and IV ($r=0.655$, $p<0.0001$) Dm values. **(C)** Moderate correlation between age at biopsy and normal mucosa *SFRP1* ($r=0.554$, $p<0.0001$), *SFRP2* ($r=0.550$, $p<0.0001$) and *SLC5A8* ($r=0.554$, $p<0.0001$) Dm values.

A.

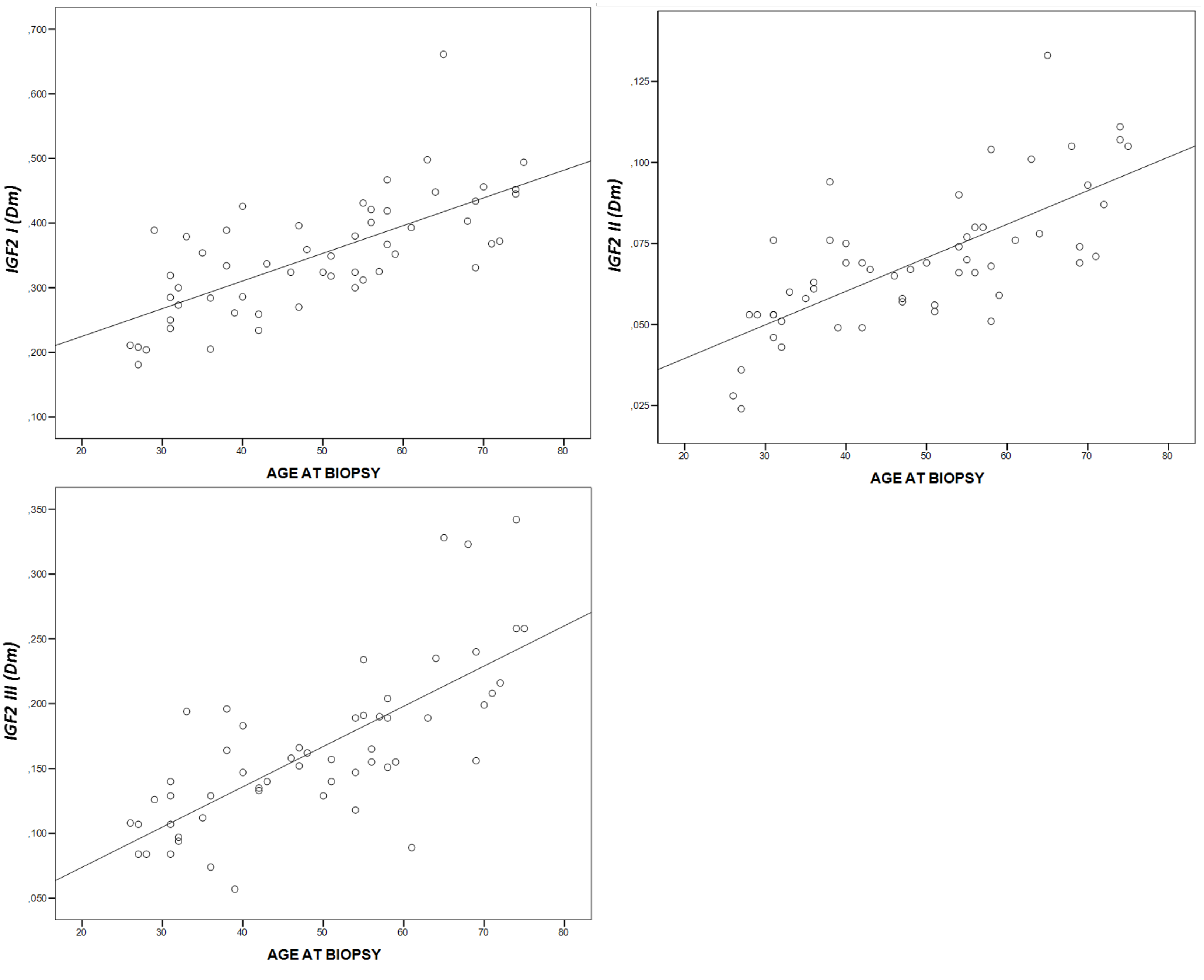

B.

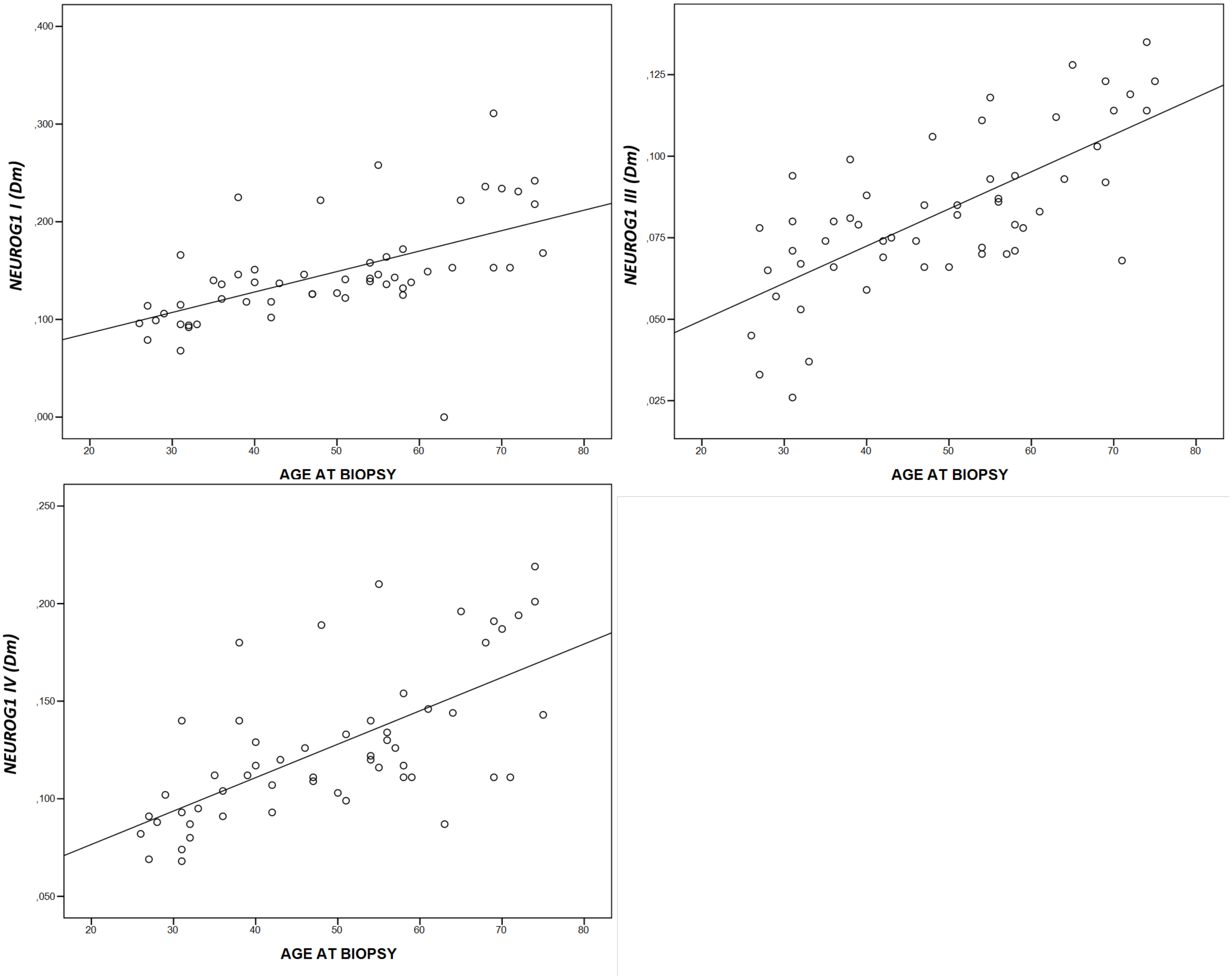

NEUROG1 I (Dm)
,400
,300
,200
,100
,000
20
30
40
50
60
70
80
AGE AT BIOPSY
NEUROG1 III (Dm)
,125
,100
,075
,050
,025
20
30
40
50
60
70
80
AGE AT BIOPSY
NEUROG1 IV (Dm)
,250
,200
,150
,100
,050
20
30
40
50
60
70
80
AGE AT BIOPSY

C.

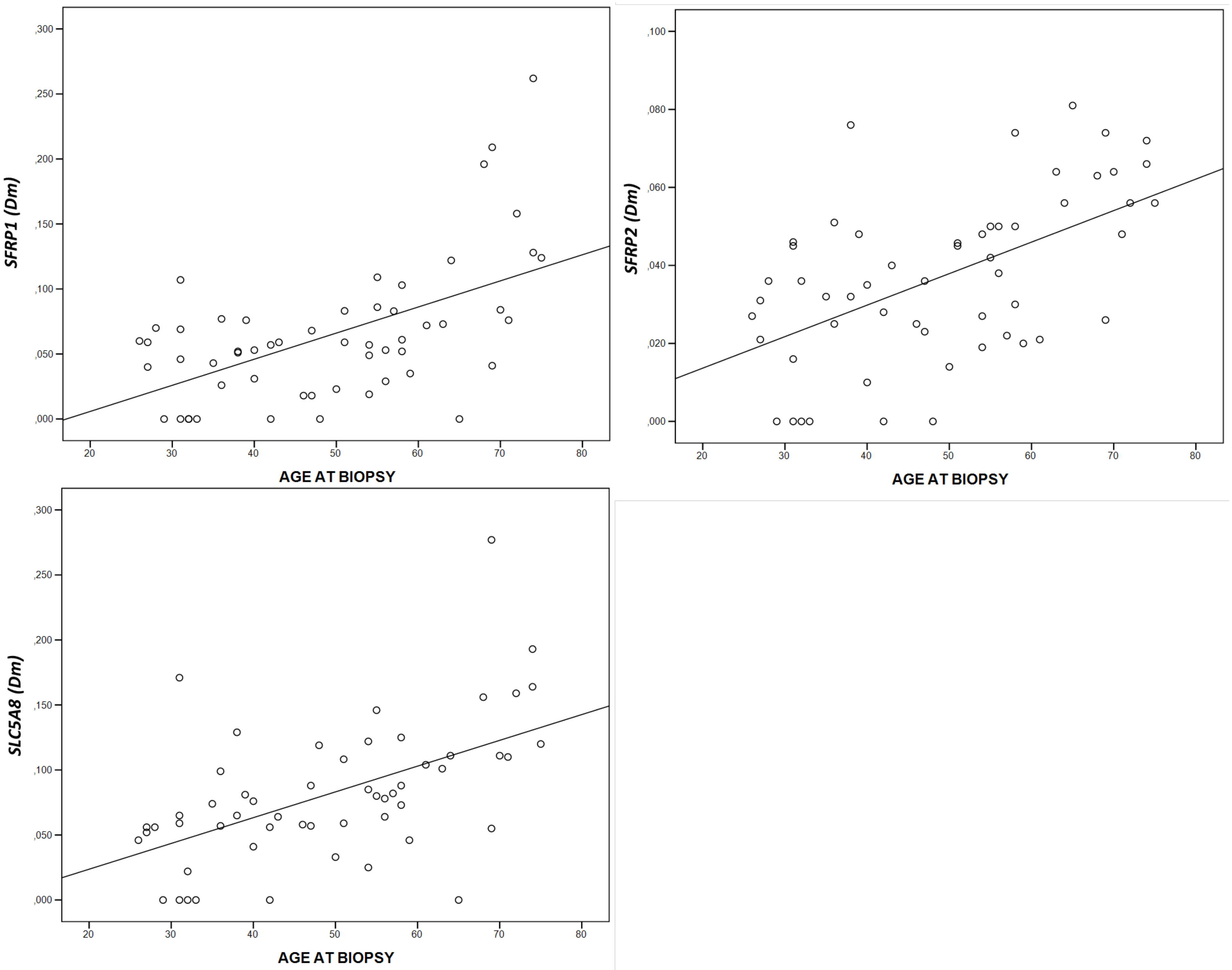
SFRP1 (Dm)
,300
,250
,200
,150
,100
,050
,000
20
30
40
50
60
70
80
AGE AT BIOPSY
SFRP2 (Dm)
,100
,080
,060
,040
,020
,000
20
30
40
50
60
70
80
AGE AT BIOPSY
SLC5A8 (Dm)
,300
,250
,200
,150
,100
,050
,000
20
30
40
50
60
70
80
AGE AT BIOPSY
