# Supplementary material for: DNA hypermethylation appears early and shows increased frequency with dysplasia in Lynch syndrome-associated colorectal adenomas and carcinomas
Source: Clin Epigenetics. 2015 Jul 22;7(1):71. doi: 10.1186/s13148-015-0102-4 (PMC4511034; doi:10.1186/s13148-015-0102-4)
Supplement: Additional file 8: Figure S4. — Effect of aging and previously diagnosed CRC on normal colonic mucosa methylation. (A) IGF2 (probes I–III). (B) NEUROG1 (probes I, III, IV, and VI). [file 13148_2015_102_MOESM8_ESM.pdf]

Suppl. Fig. 4. Effect of aging and previously diagnosed CRC on normal colonic mucosa methylation. (A) *IGF2* (probes I-III) and **(B)** *NEUROG1* (probes I,III,IV and VI) normal mucosa Dm values. Study groups: Patients 50 years old and younger with no CRC diagnosed (n=22) and CRC diagnosed (n=6) and patients over 50 years old with no CRC diagnosed (n=17) and CRC diagnosed (n=10). Statistics was performed by One-way ANOVA, post hoc test Tukey for pairwise comparisons or non-parametric test Kruskal-Wallis 1-way ANOVA (k samples), pairwise comparisons. Homogeinity of variances was tested by Levene's test and normality by Shapiro-Wilk test of normality.

A.

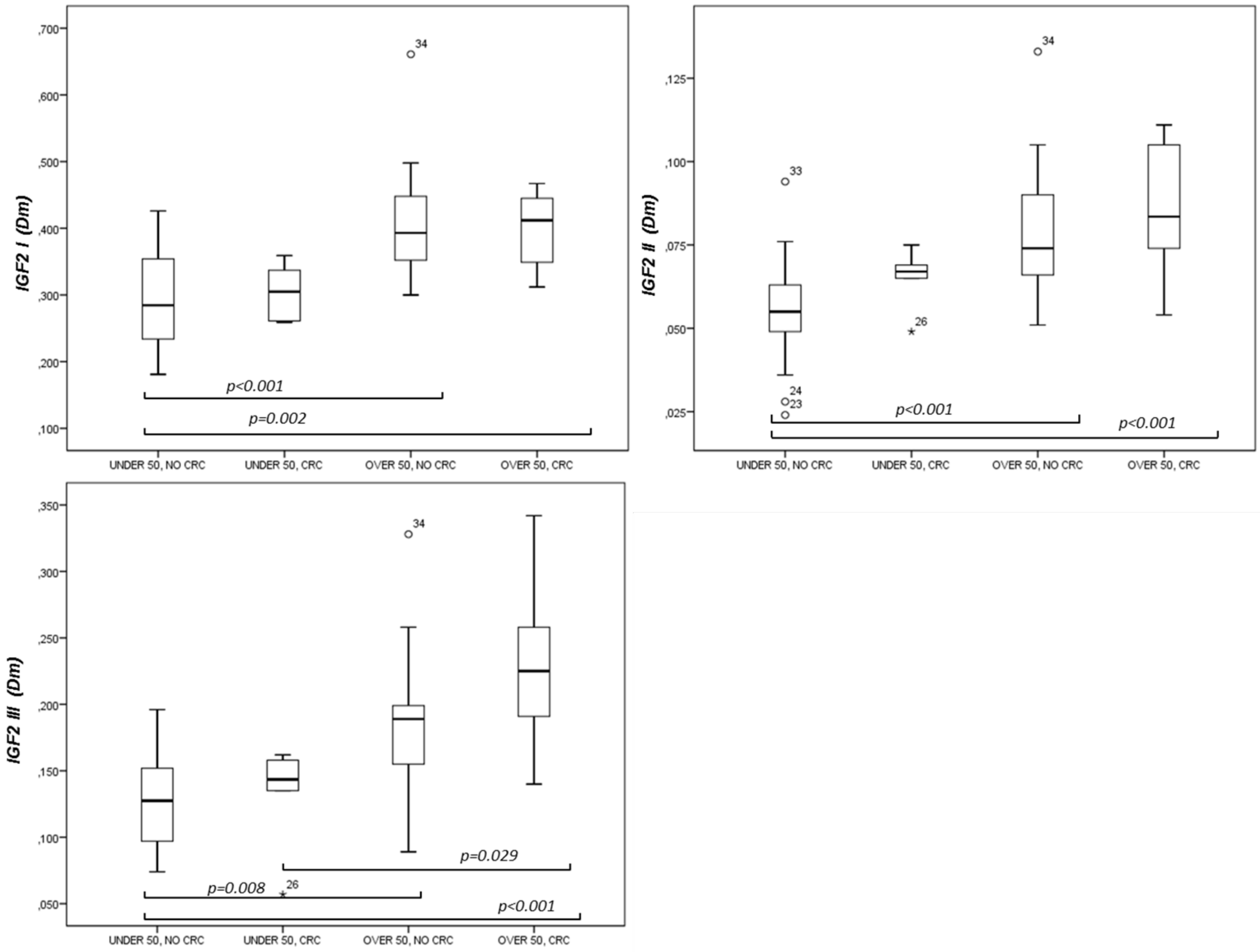

B.

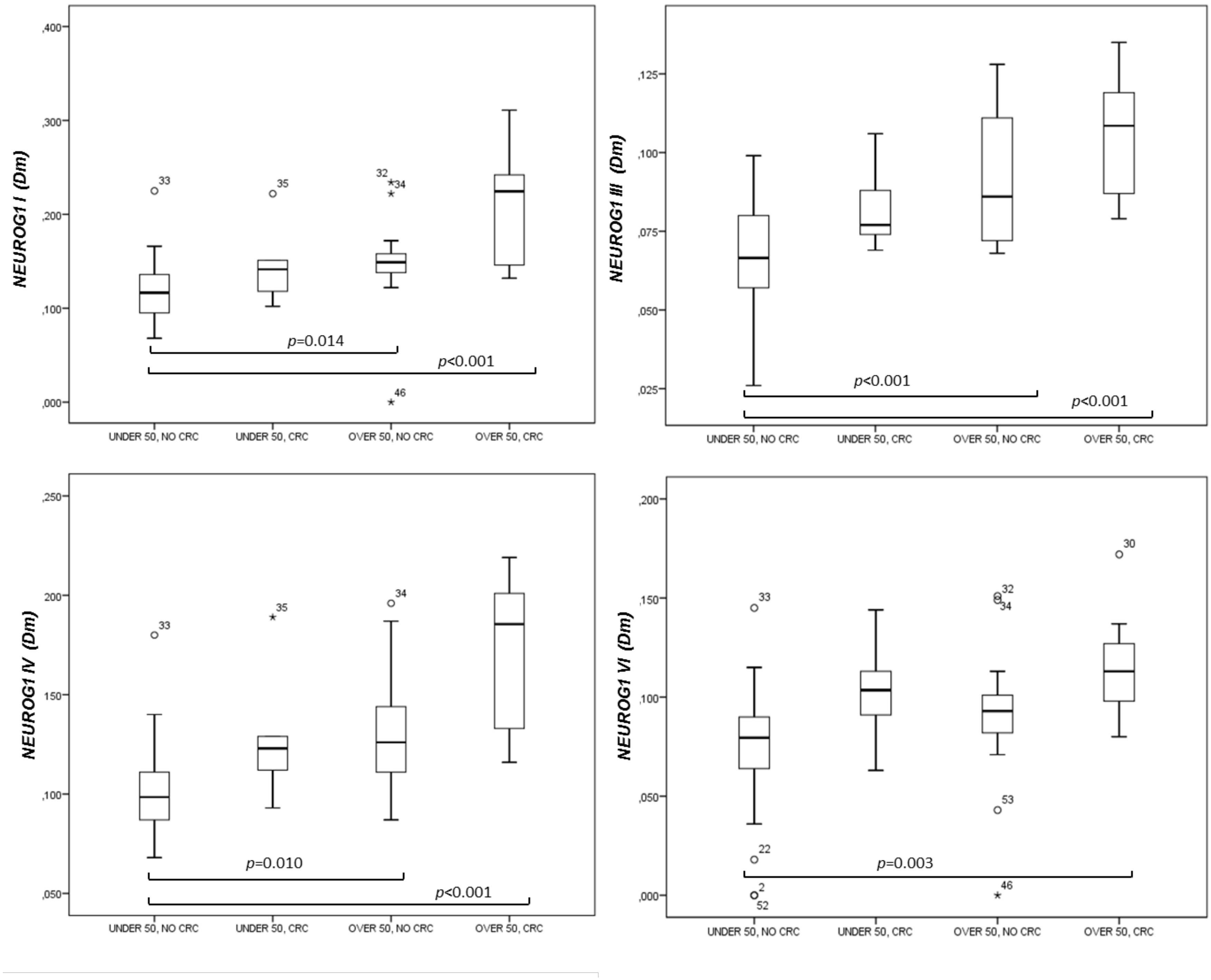
NEUROG1 I (Dm)
,400
,300
,200
,100
,000
33
35
32
34
46
p=0.014
p<0.001
UNDER 50, NO CRC
UNDER 50, CRC
OVER 50, NO CRC
OVER 50, CRC
NEUROG1 III (Dm)
,125
,100
,075
,050
,025
p<0.001
p<0.001
UNDER 50, NO CRC
UNDER 50, CRC
OVER 50, NO CRC
OVER 50, CRC
NEUROG1 IV (Dm)
,250
200
150
,100
,050
33
35
34
p=0.010
p<0.001
UNDER 50, NO CRC
UNDER 50, CRC
OVER 50, NO CRC
OVER 50, CRC
NEUROG1 VI (Dm)
,200
,150
,100
,050
,000
33
32
34
30
53
22
2
52
46
p=0.003
UNDER 50, NO CRC
UNDER 50, CRC
OVER 50, NO CRC
OVER 50, CRC
