# Supplementary material for: DNA hypermethylation appears early and shows increased frequency with dysplasia in Lynch syndrome-associated colorectal adenomas and carcinomas
Source: Clin Epigenetics. 2015 Jul 22;7(1):71. doi: 10.1186/s13148-015-0102-4 (PMC4511034; doi:10.1186/s13148-015-0102-4)
Supplement: Additional file 9: Figure S5. — MS-MLPA sensitivity for (A) CIMP and (B) candidate gene panel. Results obtained with a gradient of decreasing amount of methylated control DNA diluted into a solution of unmethylated control DNA indicate that even 5 % of methylated DNA can be reliably detected. [file 13148_2015_102_MOESM9_ESM.pdf]

**Suppl. Fig.5. MS-MLPA sensitivity for (A) CIMP and (B) Candidate panel genes.** Results obtained with a gradient of decreasing amount of methylated control DNA diluted into a solution of unmethylated control DNA indicate that even 5% of methylated DNA can be reliably detected.

**A.**

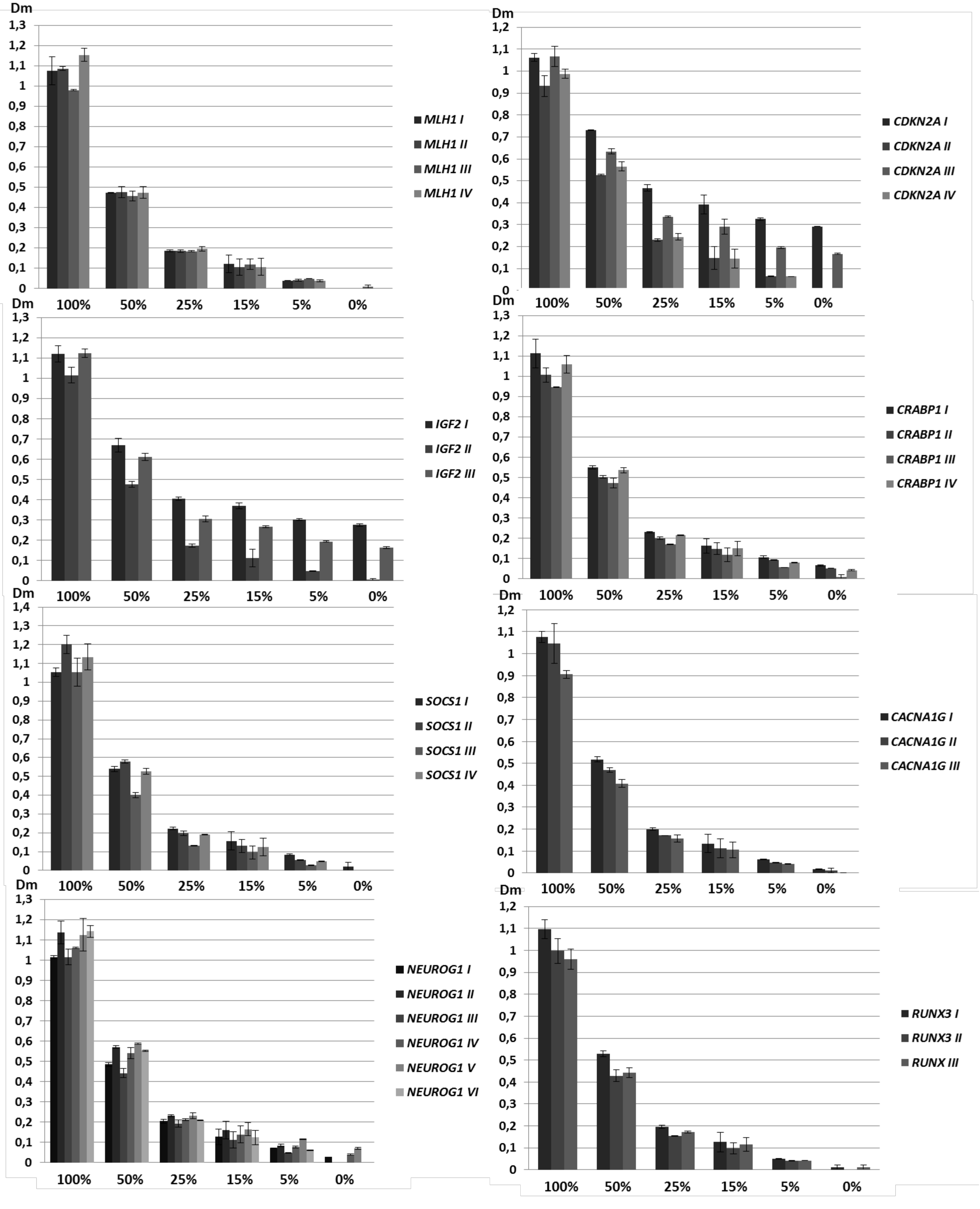

B.

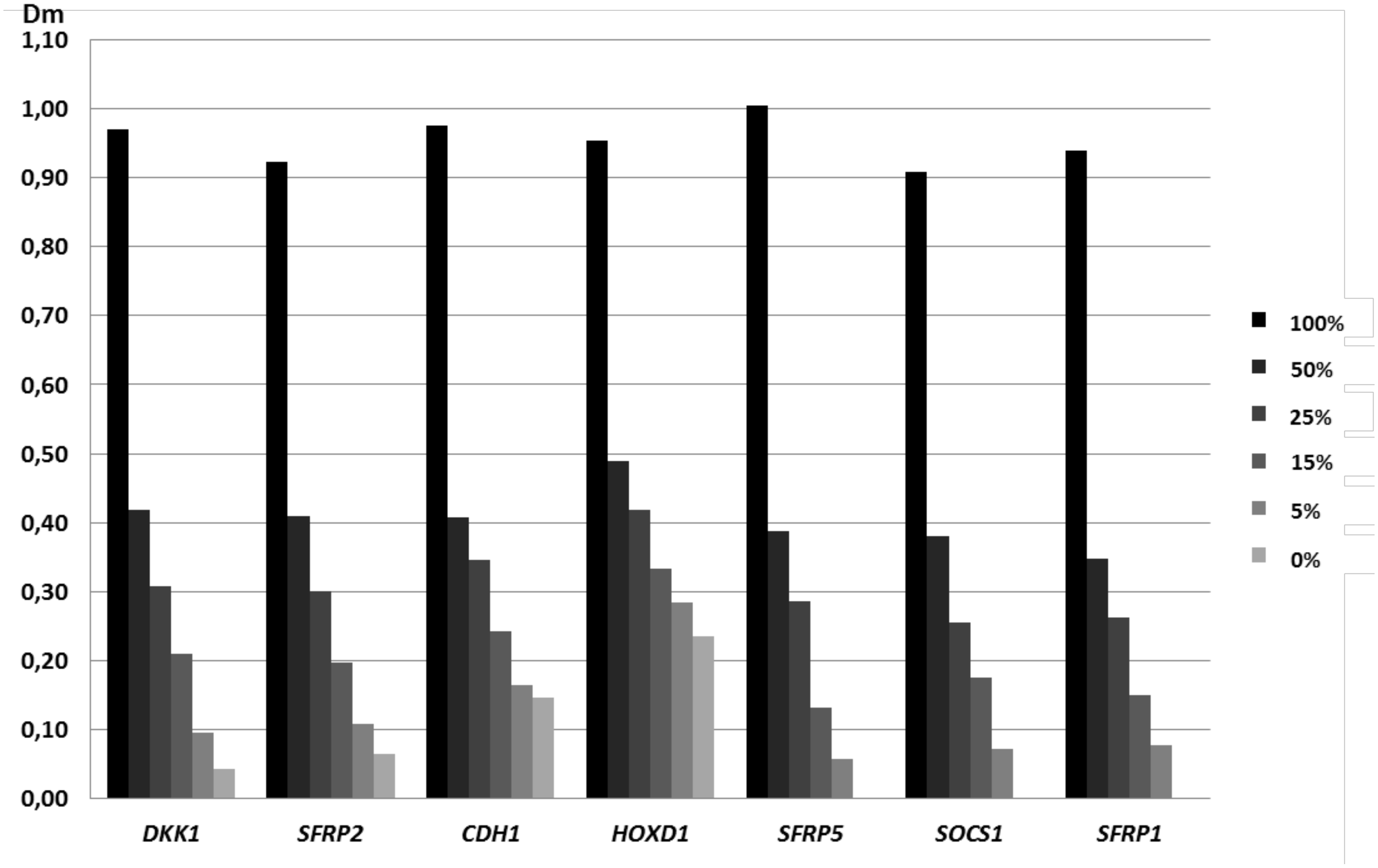

Dm
1,10
1,00
0,90
0,80
0,70
0,60
0,50
0,40
0,30
0,20
0,10
0,00
DKK1
SFRP2
CDH1
HOXD1
SFRP5
SOCS1
SFRP1
100%
50%
25%
15%
5%
0%
