# Supplementary material for: DNA hypermethylation appears early and shows increased frequency with dysplasia in Lynch syndrome-associated colorectal adenomas and carcinomas
Source: Clin Epigenetics. 2015 Jul 22;7(1):71. doi: 10.1186/s13148-015-0102-4 (PMC4511034; doi:10.1186/s13148-015-0102-4)
Supplement: Additional file 10: Table S5. — Germline mutation specifications for patients (retrospective series). [file 13148_2015_102_MOESM10_ESM.pdf]

Suppl. Table 5. Germline mutation specifications for patients included in the retrospective tumor analysis.

Mutation	Transcript	HVGS protein	No. of individuals with mutation
MLH1 c.1732-2941_1896+432del	NM_000249.3	p.Pro579_Glu633del	22
MLH1 c.454-1G>A	NM_000249.3	p.Glu153Phefs*8	5
MLH1 c.1975C>T	NM_000249.3	p.Arg659*	2
MLH1 c.546-2A>G	NM_000249.3	p.Arg182Serfs*6	1
MLH1 c.307-?_545+?del	NM_000249.3	p.Ala103Valfs*9	1
MLH1 c.543C>G	NM_000249.3	p.Gly181Gly, p.(Glu153Phefs*8)	1
MLH1 c.1039-1G>A	NM_000249.3	p.Thr347Lysfs*8	1
MSH2 c.696_697delTT	NM_000251.1	p.Ser233Hisfs*22	3
MSH2 c.1807G>A	NM_000251.1	p.Asp603Asn	2
MSH2 c.1553_1554_delCA	NM_000251.1	p.Gln518Leufs*10	1
MSH2 c.1667_1671delGACTT	NM_000251.1	p.Leu556Phefs*4	1
MSH6 c.900dupG	NM_000179.2	p.Lys301Glufs*11	1
MSH6 c.3013C>T	NM_000179.2	p.Arg1005*	1
MSH6 c.3306delT	NM_000179.2	p.Phe1104Leufs*11	1
			Total 43
